# Multicolor Photoluminescent Carbon Dots à La Carte for Biomedical Applications

**DOI:** 10.1021/acsami.3c08200

**Published:** 2023-09-16

**Authors:** Teodoro Garcia-Millan, Javier Ramos-Soriano, Mattia Ghirardello, Xia Liu, Cristina Manuela Santi, Jean-Charles Eloi, Natalie Pridmore, Robert L. Harniman, David J. Morgan, Stephen Hughes, Sean A. Davis, Thomas A. A. Oliver, Kathreena M. Kurian, M. Carmen Galan

**Affiliations:** † School of Chemistry, University of Bristol, Cantock’s Close, Bristol BS8 1TS, U.K.; ‡ Cardiff Catalysis Institute, Cardiff University, Park Place, Cardiff CF10 3AT, U.K.; § DST Innovations Ltd, Unit 6a Bridgend Business Centre, Bennett Street, Bridgend CF31 3SH, U.K.; ∥ Bristol Medical School, Public Health Sciences, Southmead Hospital, University of Bristol, Southmead Road, Bristol BS8 NB, U.K.; ⊥ HarwellXPS—The EPSRC National Facility for Photoelectron, Spectroscopy, Research Complex at Harwell (RCaH), Didcot OX11 0FA, U.K.

**Keywords:** dual fluorescence nanoprobes, co-doped carbon dots, fluorescence modulation, glioblastoma detection, bioimaging

## Abstract

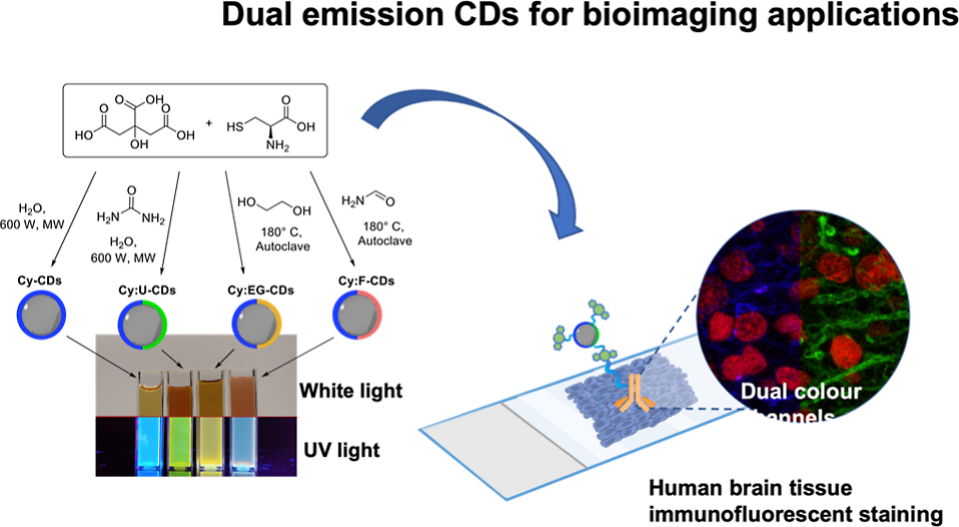

Dual-emission fluorescence probes that provide high sensitivity are key for biomedical diagnostic applications. Nontoxic carbon dots (CDs) are an emerging alternative to traditional fluorescent probes; however, robust and reproducible synthetic strategies are still needed to access materials with controlled emission profiles and improved fluorescence quantum yields (FQYs). Herein, we report a practical and general synthetic strategy to access dual-emission CDs with FQYs as high as 0.67 and green/blue, yellow/blue, or red/blue excitation-dependent emission profiles using common starting materials such as citric acid, cysteine, and co-dopants to bias the synthetic pathway. Structural and physicochemical analysis using nuclear magnetic resonance, absorbance and fluorescence spectroscopy, Fourier-transform infrared spectroscopy, and X-ray photoelectron spectroscopy in addition to transmission electron and atomic force microscopy (TEM and AFM) is used to elucidate the material’s composition which is responsible for the unique observed photoluminescence properties. Moreover, the utility of the probes is demonstrated in the clinical setting by the synthesis of green/blue emitting antibody-CD conjugates which are used for the immunohistochemical staining of human brain tissues of glioblastoma patients, showing detection under two different emission channels.

## Introduction

Functional materials based on luminescent nanoparticles have recently attracted much attention due to their unique physicochemical properties. In addition, their large surface area allows the attachment of targeting molecules and thus these types of materials have found their use in a wide range of biological and medical applications.^1,2^

Among the photoluminescent nanoparticles, carbon dots (CDs) represent a novel class of carbon-based fluorescent nanomaterials which have generated significant interest due to their chemical stability, simplicity of preparation, high water solubility, ease of functionalization, biocompatibility, and low synthetic cost.^3−7^ These quasi-spherical nanomaterials combine several attributes of semiconductor inorganic quantum dots such as broadband excitation spectra,^8^ tunable fluorescence depending on composition,^9,10^ and their <10 nm particle size which make them suitable for use in many biological application including bioimaging. Indeed, CDs have found a growing number of applications across many scientific areas such as catalysis,^11^ sensing,^12^ and functional materials.^13^ CDs are particularly valuable as antimicrobial agents,^14^ in gene delivery,^15−18^ cell imaging,^19,20^ in vitro theranostics,^21−23^ photosynthesis augmentation,^24−26^ cancer sensing,^27^ and photocatalysis,^28−30^ among others.

However, despite much progress in the development of synthetic strategies to access this nanomaterial,^31−34^ there are many remaining challenges including the lack of reproducibility between approaches due to the inherent heterogeneity of the generated materials, the fact that small variations in the synthetic conditions can lead to completely different nanomaterials,^35^ and a lack of fundamental understanding at a molecular level of their mechanism of formation, chemical structure, and how these parameters correlate to their photoluminescence (PL) properties.

It has been shown that the structural and morphological properties of CDs can help regulate the chemical stability and fluorescence quantum efficiencies of the nanomaterials. Indeed, previous studies have established that high fluorescence quantum yields (FQYs) in CDs are ascribed to the limitation of intramolecular motion of fluorophores within a rigid structure (*e.g.*, the core of a CD).^36,37^ These features can delay the thermalization of photoexcited states, which otherwise quenches the fluorescence. Another investigation has suggested that high FQYs are associated with nitrogen-enriched chemical groups which introduce trap N-states and facilitate the radiative recombination.^38^ Moreover, the modulation of the dominant fluorescence is often attributed to surface motifs,^39^ these groups can introduce an additional manifold of states (mid-gap states) below the conduction band, adjusting the absorption and emission wavelength of the blue fluorescence.^40−47^ It is generally agreed that the hydrothermal synthesis of citric acid (CA)-based CDs under continuous heating proceeds via the polymerization of the small molecule carbon sources, followed by carbonization and subsequent aromatization and surface passivation. However, how chemical structure correlates with fluorescence emissions has been much more difficult to establish. In this context, Qu et al.,^48^ contrary to other reports, suggested that large-size conjugated domains are the basis for the red-shift in the band gap emission and that the PL quantum efficiencies are dependent on the charges present on the CD surface. In parallel, Hola et al.^49^ noted that the red-shifted emission in CDs derived from CA originates from a high content of graphitic N and is independent of the degree of aromatization. Later, Li and co-workers studied the modulation of the band gap fluorescence of similar CDs synthesized from CA and urea, suggesting that the primary origin of the long-wavelength emission arises from electron-acceptor moieties, rich in sulfoxide/carbonyl groups bound to the outer layers that promote radiative relaxations in the red spectral region.^47^ Overall, several lines of evidence indicate that multiple factors coexist and affect the emission of the resulting nanoparticles.^50^ Thus, despite recent progress, there are still gaps in our current fundamental understanding of CD fluorescence modulation, and most CD syntheses are a result of serendipity, rather than rational design tailored to bespoke applications.

In terms of biomedical applications, there is an urgent need to develop low-cost sensitive tests for the detection of brain tumors to help general practitioners in primary care.^51^ The most common malignant primary brain tumor called glioblastoma (GBM) is characterized by abnormal blood vessels resulting in a leaky Blood–Brain Barrier.^52,53^ Glial Fibrillary Acid Protein (GFAP) is unique to the brain and not present in normal peripheral blood. Antibodies targeting GFAP are used to diagnose gliomas in tissue samples. We recently reported a practical and general method for the labeling of proteins and antibodies with blue emissive fluorescent CDs to generate antibody (Ab)-CD probes that could be used for the successful immunohistochemical staining of human brain tissues of patients with GBM.^54^ However, the inherent autofluorescence of many tissues and cells can sometimes be problematic for the detection of a particular exogenous fluorophore such as blue-emitting CDs.^55^ In this context, dual-emission fluorescent probes are particularly attractive in comparison to those with a single emission center since these types of nanomaterials can help improve the sensitivity of detection and minimize background interference using dual imaging channels^56−58^ and, therefore, such probes will be essential to improving clinical diagnostics.

Herein, we describe a practical general strategy for the synthesis of multicolor emission CDs with high FQYs using two common and inexpensive starting materials. The facile-to-reproduce method uses cost-effective precursors such as CA and cysteine (Cy) giving rise to bright-emitting CDs. Color tunability is achieved by the addition of commercial co-precursors during the synthetic protocol assisted by either MW irradiation or hydrothermal treatment, yielding CDs with dual emission profiles (e.g., green/blue, yellow/blue, or red/blue). The resulting strategy provides a robust synthetic pathway toward CDs with defined compositions and physicochemical properties that could be attributable to fluorescence modulation. The versatility of these novel nanomaterials in clinical applications is demonstrated in the application of green/blue-emitting Ab-CD conjugates for the immunohistochemical staining of human brain tissues of patients with GBM.

## Results and Discussion

**Scheme 1:**
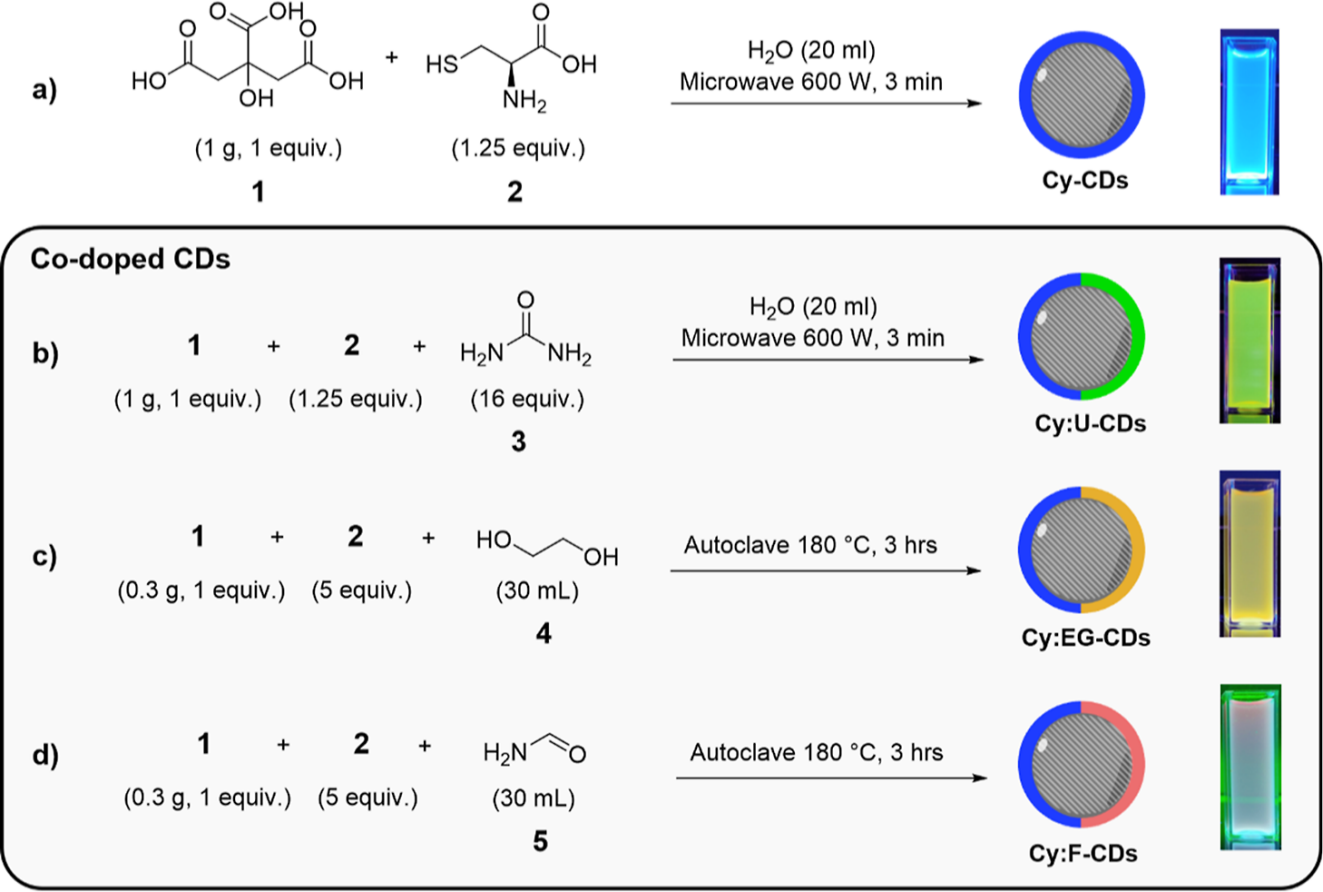
General Synthetic Preparation of (a) **Cy-CDs**, (b) **Cy:U-CDs**, (c) **Cy:EG-CDs**, and (d) **Cy:F-CDs** Digital images of CD solutions irradiated with 380 nm light for **Cy-CDs** and **Cy:U-CDs**, polychromatic UV light for **Cy:EG-CDs** and combined polychromatic UV and green wavelength irradiation for **Cy:F-CDs**.

Following our previous efforts on the MW-assisted synthesis of CDs using carbohydrates,^59,60^ and based on the evidence of earlier reports using citric acid (CA) and cysteine (Cy) starting materials,^61^ the synthesis of bright blue-emitting fluorescent CDs (**Cy-CDs**) started by using a 1:1.25 ratio of CA **1** (as the major carbon precursor) and Cy **2** (as the nitrogen/sulfur-containing precursor) in water, and a 3 min MW irradiation-assisted method (600 W) to ensure the carbonization of the carbogenic centers (Scheme 1a). In this approach, **1** was chosen since it includes a triple-branched carboxyl feature which facilitates the polymerization of reaction intermediates, whereas **2** contains the required nucleophilic amine and thiol to facilitate the initial intramolecular condensation with **1** required for CD formation. Indeed, it has been shown that after the formation of molecular intermediates akin to 2-pyridone, neighboring carboxylic functionalities can carry out simultaneous intramolecular condensation steps for the polymerization of molecular derivatives.^62,63^

**Figure 1:**
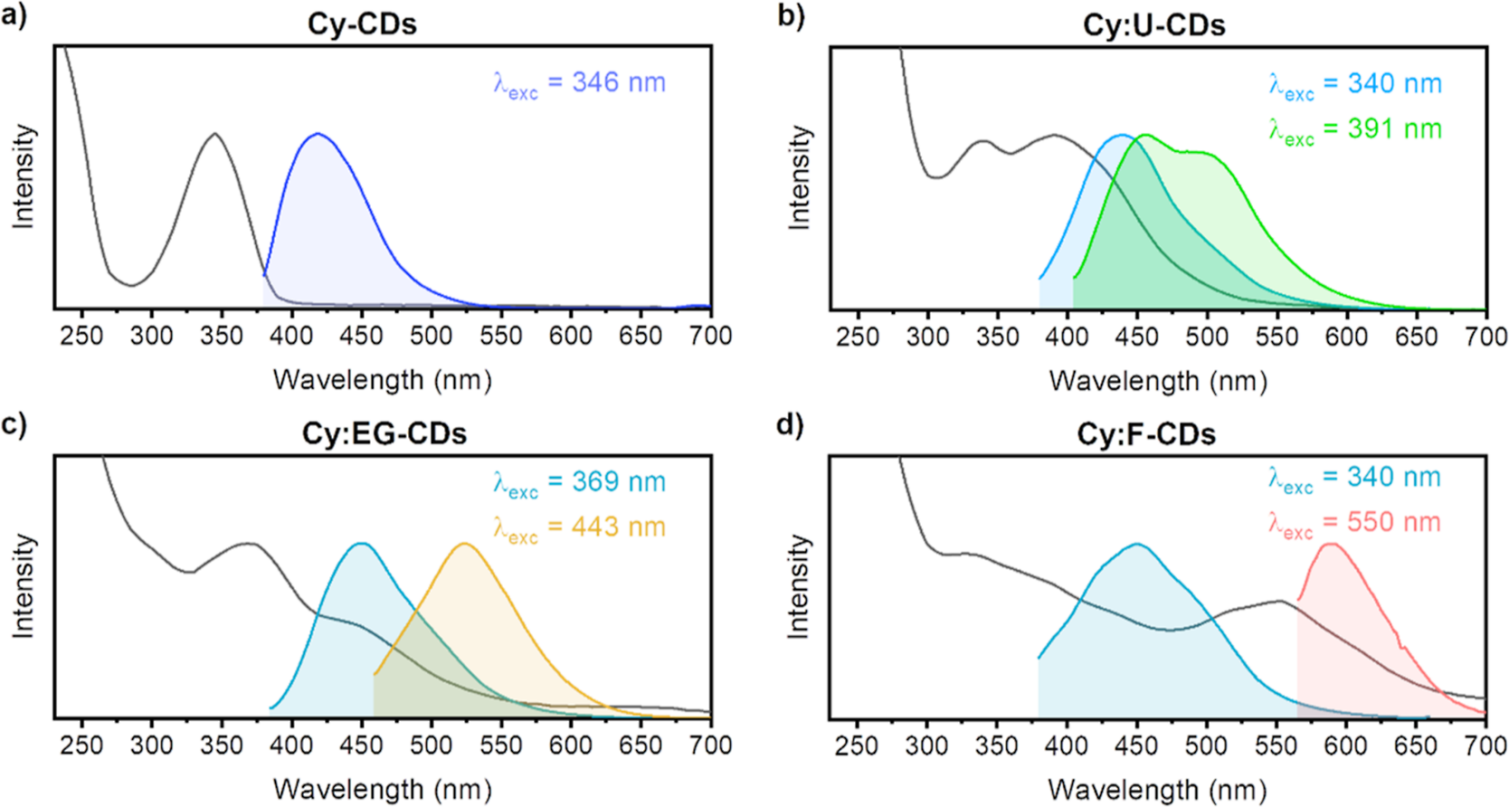
UV Absorption (black) and fluorescence (color) spectra of CDs for different excitation wavelengths: (a) **Cy-CDs**, (b) **Cy:U-CDs**, (c) **Cy:EG-CDs**, and (d) **Cy:F-CDs**.

Monitoring the reaction with ^1^H nuclear magnetic resonance (NMR) at different irradiation times showed that a molecular fluorophore identified as 5-oxo-2,3-dihydro-5*H*-(1,3)thiazolo[3,2-*a*]pyridine-3,7-dicarboxylic acid (TPA, Figures S1–S8) is the major component of **Cy-CDs**, as evidenced by the characteristic resonances at δ 6.7, 5.5, 3.8 and 3.6 ppm (Figure S9).^64^ Furthermore, fluorescence spectroscopy analysis comparing the emission profiles of TPA and **Cy-CDs** demonstrated that the blue emission could be attributed to the presence of TPA molecules on the **Cy-CDs**’ structure (Figure 1a and S10). These results are in agreement with previous reports where it was found analogous organic fluorophores such as 5-oxo-3,5-dihydro-2*H*-thiazolo[3,2-*a*]pyridine-3,7-dicarboxylic acid (TPDCA) and 5-oxo-3,5-dihydro-2*H*-thiazolo[3,2-*a*]pyridine-7-carboxylic acid (TPCA) were the main source and fluorescence origins of other N,S-CDs.^65^

Having achieved bright fluorescent CDs, the tuneability of the fluorescence wavelengths was explored by the addition of a series of co-precursors such as urea (U) **3**, ethylene glycol (EG) **4**, or formamide (F) **5** to the optimized synthetic protocol (Scheme 1b–d). The co-precursors were chosen to include additional surface and core functionalities capable of tuning the emission wavelength of the resulting products.^66,67^ To that end, an excess of **3** (16 equiv) in combination with **1** and **2** under microwave irradiation yielded green/blue emissive nanoparticles **Cy:U-CDs**. Similarly, it was possible to tune the fluorescence of the CDs to either yellow/blue (**Cy:EG-CDs**) or red/blue emission (**Cy:F-CDs**) by replacing water as the solvent with **4** or **5**, respectively. Under these conditions, both solvents also act as co-doping agents; however, their high boiling points prevented the reaction solvent from evaporating using the MW protocol, which may partly explain the low product yields obtained in the synthesis of CDs using the irradiation-assisted protocol in the presence of **4** or **5** as solvents. As a result, an optimized protocol was devised for the synthesis of **Cy:EG-CDs** and **Cy:F-CDs** whereby an autoclave reactor was used instead to achieve the desired product. Important to note that an excess of the nucleophile component, e.g., Cysteine was important to obtain different emission profiles. This is likely due to changes in the reaction pathway which in turn leads to the generation of different fluorescent centers and an overall distinct emission profile.^35^ The formation of nanoparticles using our synthetic protocols was confirmed by XRD, TEM, and AFM (Figures S11–S14). XRD analysis confirmed that **Cy:EG-CDs** and **Cy:F-CDs** displayed crystalline peaks which did not correspond to the starting materials l-cysteine and citric acid (Figure S11). Moreover, TEM studies of all four samples showed that all CDs were typically less than 10 nm in diameter (Figure S12). Samples **Cy:EG-CDs** and **Cy-CDs** were stable to high-resolution imaging and interplanar spacings of ∼2.55 and ∼2.21 Å were determined, respectively (Figure S13). It has not been possible to obtain interplanar spacings from samples **Cy:U-CDs** and **Cy:F-CDs** currently.

Unlike with **Cy-CDs**, NMR analysis revealed the presence of nitrogen-containing aromatic species different from TPA, as the major components in co-doped CDs, as evidenced by ^1^H NMR (**Cy:U-CDs** at δ 8.34 (s), 7.94 (s), 6.61 (m), and 6.47 (m) ppm; **Cy:EG-CDs** at δ 7.73 (s), 7.47 (s) and 6.69 (s) ppm and, **Cy:F-CDs** at δ 8.32 (s) and 7.92 (s) ppm; see Figures S15–S17), which suggest that the synthesis of co-doped CDs must undergo alternative synthetic pathways during CD formation. The chemical features that distinguish co-doped CDs from **Cy-CDs** are accompanied by excitation wavelength-dependent emission for green-to-red multicolor fluorescence (see overview in Figure 1), which is not present in **Cy-CDs**. This excitation-dependent behavior indicates the presence of multiple photoluminescence centers (PLCs) and has been reported for other CDs.^40^ It is important to note that diffusion-ordered NMR spectroscopy of all CD samples confirmed that the materials diffuse as a single entity and strongly supports the conclusion that observed fluorescent features are not due to unconjugated molecular fluorophores^65^ (Figures S18–S21).

Absorption spectroscopy was used to establish the major electronic absorption bands associated with the different CD materials generated under the different synthetic protocols (Figure 1). A very strong absorption band at shorter wavelengths than 270 nm was evident in the absorption spectra of all CDs synthesized. Between 300 and 370 nm, these spectra contain secondary absorption maxima at 346 nm (**Cy-CDs**), 340 nm (**Cy:U-CDs** and **Cy:F-CDs**), and 369 nm (**Cy:EG-CDs**). The observed shift in this maxima could be attributed to variations in heteroatom functionalities between CDs.^68^ A third absorption band was evident with co-dopants, *e.g.*, co-dopant **3** (**Cy:U-CDs**), gave rise to a lower-energy absorption band with a maximum centered at 391 nm. Similarly, **Cy:EG-CDs** showed a separate absorption feature associated with the shoulder centered at 443 nm. The absorption spectrum of **Cy:F-CDs** was notably less well defined and broader than other CDs, exhibiting appreciable absorbance through most UV and visible wavelengths, with a third absorption maximum centered at 550 nm. The breadth of absorption bands in co-doped CDs is associated with the structural heterogeneity of superimposing optical centers.^69^

**Figure 2:**
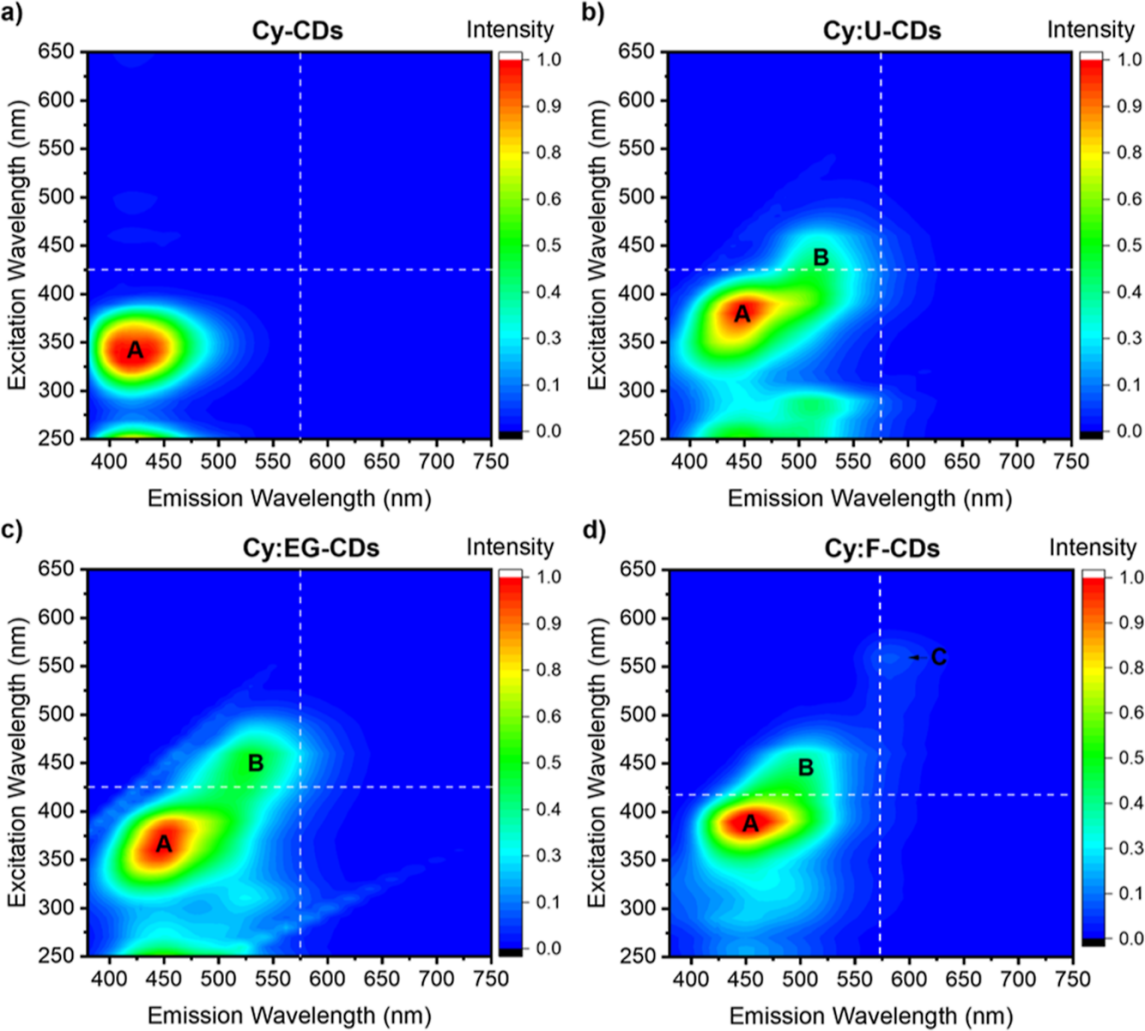
Two-dimensional fluorescence spectra of (a) **Cy-CDs**, (b) **Cy:U-CDs**, (c) **Cy:EG-CDs**, and (d) **Cy:F-CDs** at 0.4 mg/mL. Fluorescent bands A, B, and C are assigned separately for each CD.

In turn, fluorescence spectroscopy was used to help identify the number of radiative states associated with each CD generated. Analysis of the fluorescence spectra revealed that the deep-blue emission for **Cy-CDs** located at 418 nm correlates to the emission maximum of TPA (Figure S10A,B excitation spectra of CDs for different emission wavelengths). For **Cy:U-CDs**, **Cy:EG-CDs**, and **Cy:F-CDs** the blue emission maxima shifted to ∼450 nm. Closer inspection of the two-dimensional fluorescence spectra revealed that the radiative decay of **Cy-CDs** (Figure 2a) was also distinguished from co-doped CDs by presenting excitation-independent (*e.g.*, Kasha-type) behavior, hence, homogeneous in nature and likely associated with molecular TPA states. Nonetheless, for **Cy:U-CDs**, **Cy:EG-CDs**, and **Cy:F-CDs**, excitation-dependent emission throughout the “blue” region of the spectrum (labeled A in Figure 2) correlates to variable energies for the radiative decay of multiple photoluminescent centers. It seems possible that isolated sp^2^ domains are the origin of the blue emission and that the effect of structural heterogeneity gives rise to multiple inter-band states which results in the excitation-dependent behavior.^49,50,63,70,71^ Further, the lower-energy absorption regions in co-doped CDs give rise to entirely separate red-shifted fluorescence bands (appearing close to the “diagonal”), indicative of distinct additional fluorophores within the nanomaterials. Thus, the blue emission A is accompanied by the fluorescence of polyaromatic fluorophores (bands B and C) at longer excitation wavelengths.^49,63^ The observed green or yellow fluorescence in **Cy:U-CDs**, **Cy:EG-CDs**, and **Cy:F-CDs** corresponded to emission maxima at 499, 523, and 489 nm, respectively. In the case of **Cy:U-CDs** and **Cy:F-CDs**, the excitation-dependent behavior of band A overlaps with most part of the green emission (band B). Whereas in **Cy:EG-CDs**, the shift of the B band separates its yellow emission from the blue band. The red-shifted fluorescence in **Cy:F-CDs** (band C) is also entirely isolated due to its dramatic red-shift in the 2D fluorescence spectrum (maximum at 590 nm).

In order to study the effect of CD structural changes upon the fluorescence quantum efficiencies of the different CD samples, the FQYs of the major fluorescent bands listed above (Figure 2a–c) were measured (Table S1). The excitation-independent emission in **Cy-CDs** exhibits a total integrated FQY of 0.67 (Figure S22a). However, the incorporation of **3** within the synthesis (**Cy:U-CDs**) or the use of **4** or **5** as solvent/co-dopants under autoclave-assisted conditions (**Cy:EG-CDs** and **Cy:F-CDs**) introduces additional fluorescence bands but also decreases the FQY of band A to 0.15, 0.10, and 0.23, respectively (Figure S22b–d). The fluorescence of band A in co-doped CDs likely originates from hybridized sp^2^ domains; thus, it is possible that synthetic pathways leading to the hybridization of additional functional groups directly impact the FQY. Groups which modulate the excitation-dependent fluorescence introduce new absorption bands, cf. **Cy-CDs** (see Figure 1) that have different associated radiative pathways with variable quantum efficiencies. Moreover, the hybridization of oxidized groups (*e.g.*, carbonyl, epoxide among other groups), which instead acts as nonradiative electron–hole recombination centers, is likely to decrease the FQY.^40,72,73^ On the other hand, the FQYs specific for the B band fluorescence which is only present in **Cy:U-CDs**, **Cy:EG-CDs**, and **Cy:F-CDs** were found to be 0.22, 0.13, and 0.15, respectively (Figure S23a–c), whereas the FQY specific for the red-emissive band C in **Cy:F-CDs** was determined to be 0.44 (Figure S23d). Even with the different starting materials used, and resulting shifted fluorescence bands, the total FQYs (e.g., integrating over bands A–C) remain high (>0.43) across the reported series of CDs.

**Figure 3:**
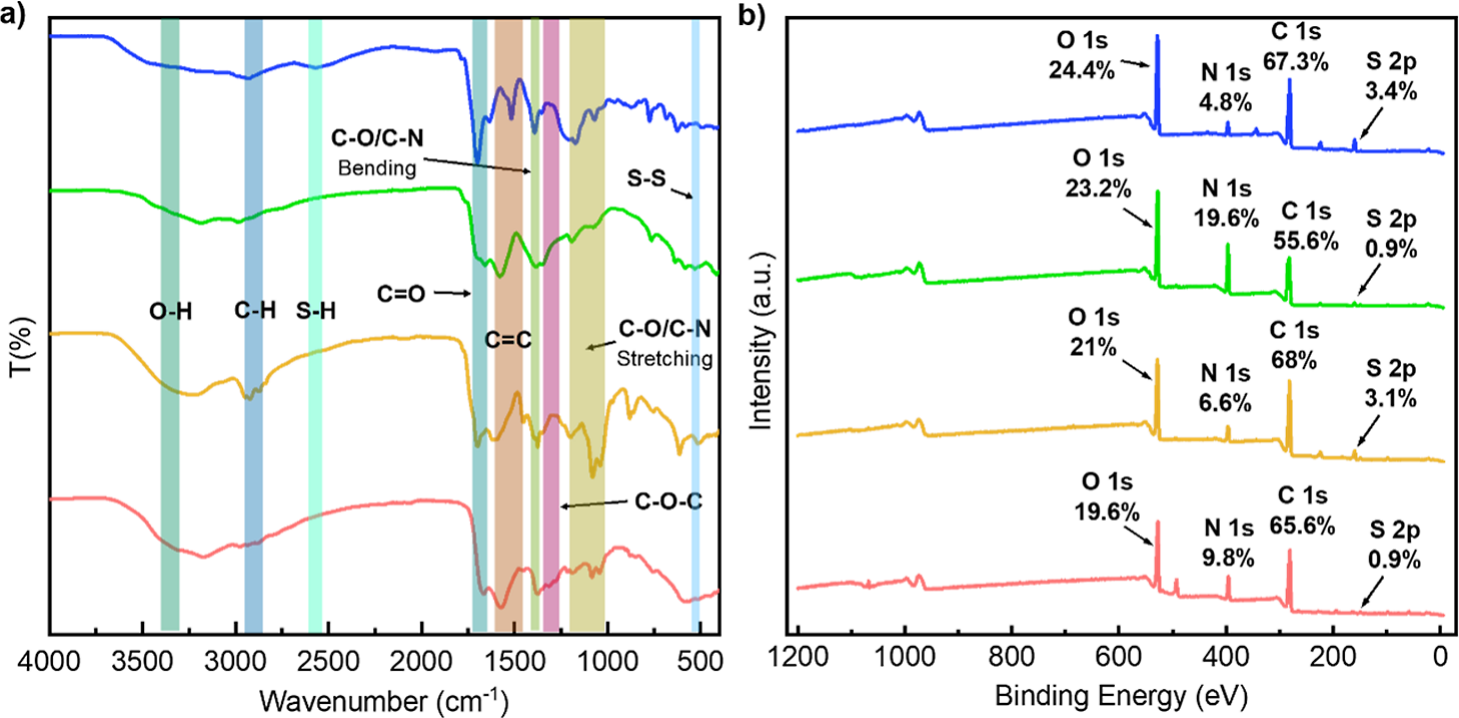
FT-IR spectra (a) and XPS surveys (b) of **Cy-CDs**, **Cy:U-CDs**, **Cy:EG-CDs**, and **Cy:F-CDs** inserted from top to bottom, respectively. Elemental composition (%) of S, C, N, and O heteroatoms is included in the XPS analysis.

To gain further insights into the surface functionalization of the different CD samples, Fourier-transform infrared spectroscopy (FTIR) and X-ray photoelectron spectroscopy (XPS) analyses were undertaken (Figure 3). The presence of C=C bonds (1600–1450 cm^–1^) in all samples, typical of the skeletal aromatic structure in CDs, was confirmed by FTIR spectroscopy. Characteristic bands for carboxyl C=O and C–O/C–N (bending) groups were also observed between 1720–1650 and at 1400 cm^–1^, respectively for all CDs (Figure 3a). Signals related to O–H, C–H, and C–O/C–N (stretching) at 3400–3350, 2950–2850, and 1200–1010 cm^–1^, respectively, were more pronounced for **Cy:EG-CDs** and **Cy:F-CDs**. However, **Cy:F-CDs** displayed a significant contribution from vibrations associated with C–O–C groups, as evidenced by the bands between 1335 and 1250 cm^–1^. Thiol moieties correlating to the S–H stretching bands observed between 2550 and 2600 cm^–1^ were detected for **Cy-CDs**, while a weak signal associated with a disulfide (S–S) vibration was observed at 500–540 cm^–1^ for **Cy:EG-CDs**.

XPS analysis demonstrated that the CDs contained C, N, O, and S elements and that the heteroatom composition was strongly dependent on the synthetic conditions (Figure 3b). In **Cy-CDs**, the N/S composition remains approximately similar, however, following the addition of co-dopants **3**, **4**, and **5**, an increase in the N % content within the CDs is observed with a 4.1-, 1.4- and 2-fold increase for **Cy:U-CDs**, **Cy:EG-CDs**, and **Cy:F-CDs**, respectively. It was also found that using co-dopants **3** or **5** (as in **Cy:U-CDs** and **Cy:F-CDs**) leads to a decrease in the S content, which suggests that the addition of co-dopant agents can bias the reaction pathway by competing for the available electrophilic centers, *e.g.*, citric acid. On the other hand, using **4** or **5** as solvent/co-dopant (**Cy:EG-CDs** or **Cy:F-CDs**) under autoclave conditions unexpectedly increased the C to O ratio which might be the result of using a different heating method. It is interesting to note that the S content in **Cy:EG-CDs** is similar to that of **Cy-CDs**, suggesting that **2** is still able to dope the composition of the CDs to a similar extent.

**Figure 4:**
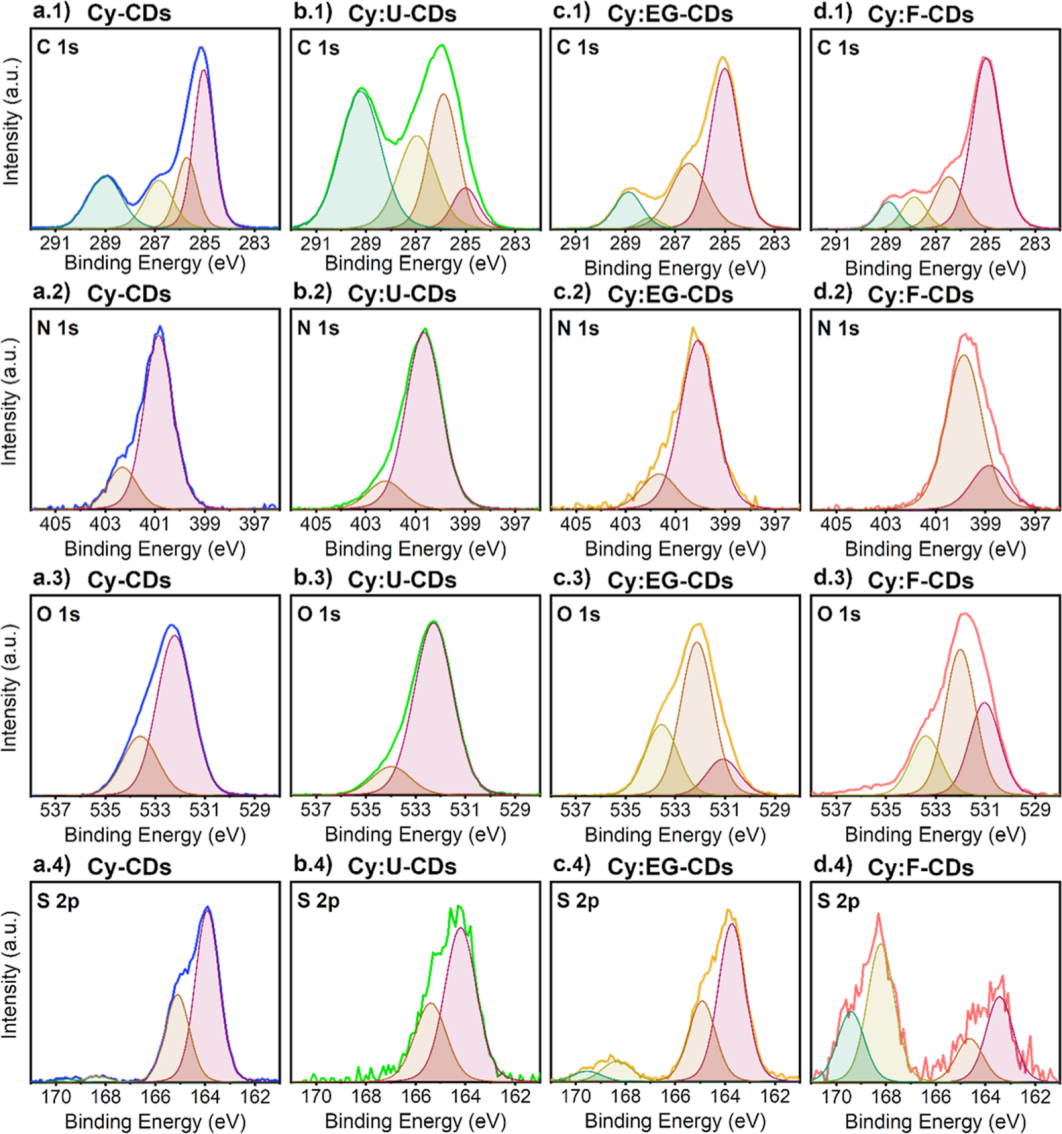
High-resolution XPS spectra of CDs. Survey for **Cy-CDs**, **Cy:U-CDs**, **Cy:EG-CDs**, and **Cy:F-CDs** included in columns a, b, c, and d, with deconvoluted bands for narrow C 1s, N 1s, O 1s, and S 2p scans, in rows 1, 2, 3 and 4, respectively.

To further interrogate the chemical and functional group composition of CDs was investigated with high-resolution XPS (Tables S2–S5, Supporting Information for further details). In the case of **Cy-CDs**, the narrow C 1s spectrum was fitted to four peaks at 285.0, 285.7, 286.8 and 289.0 eV which can be attributed to C–C/C=C, C–COO/S–C, N–C/O–C and O=C/O–C=O, respectively (Figure 4, row 1). The N 1s spectrum has two signals at 400.8 and 402.3 eV correlated to amine/pyrrolic N and imide/graphite N (Figure 4, row 2). Conversely, the O 1s peak is deconvoluted into two broad bands at 532.2 and 533.6 eV, confirming the presence of C=O/O–C=O/C–OH/C–O–C and O=C–O (Figure 4, row 3), while in the S 2p spectrum, the binding energies of 163.9, 165.1, 168.3, and 169.5 eV reveal the presence of −C–S– (2p_3/2_, thiophene), −C–S– (2p_1/2_, thiophene), C–SO_2_–/C–SO_3_– and sulfate groups, respectively (Figure 4, row 4).

### Anti-GFAP Ab-CDs Immunostaining of Clinical Glioblastoma Tissue

GFAP immunostaining with fluorescently labeled proteins is the most commonly used method to examine the distribution of astrocytes and the hypertrophy of astrocytes in response to neural degeneration or injury as in the development of GBM.^74^

Traditional strategies employ molecular dyes, which tend to be expensive and subject to photobleaching. Alternatively, fluorescent nanoparticles can be tuned to exhibit high stability, sensitivity and specificity for their desired target without the limitations of organic fluorophores and thus have found many applications as robust probes in many bioimaging and diagnostic applications.^75^ We have recently reported a protocol for the generation of blue-emitting CDs-antibody conjugates to be used for the immunohistochemical staining of human brain tissue of patients diagnosed with glioblastoma.^54^ However, the CDs blue emission (450 nm), which is in the same range as the cell autofluorescence, can affect the sensitivity of the assay, and thus probes with emission profiles outside this range are highly desirable. Having now access to CDs with dual emission, we decided to explore the feasibility of using the newly developed CDs in a clinical application. Dual-emission fluorescent probes are particularly useful as more sensitive probes for biomedical applications since the interference of the detection background can be avoided. Indeed, ratio-labelling is now possible since identification of the probe can be made on the basis of their color intensity ratio and not on the color composition alone, which can avoid artifacts arising from cell/tissue autofluorescence.^76^ We thus hypothesize that dual emission fluorescence bands present on the new CDs could be exploited for the detection of GFAP in brain tissues of cancer patients where simultaneous detection of two distinct emission channels could be used for detection and to exemplify this, **Cy:U-CDs** which emits in the blue and green channels was selected.

**Scheme 2:**
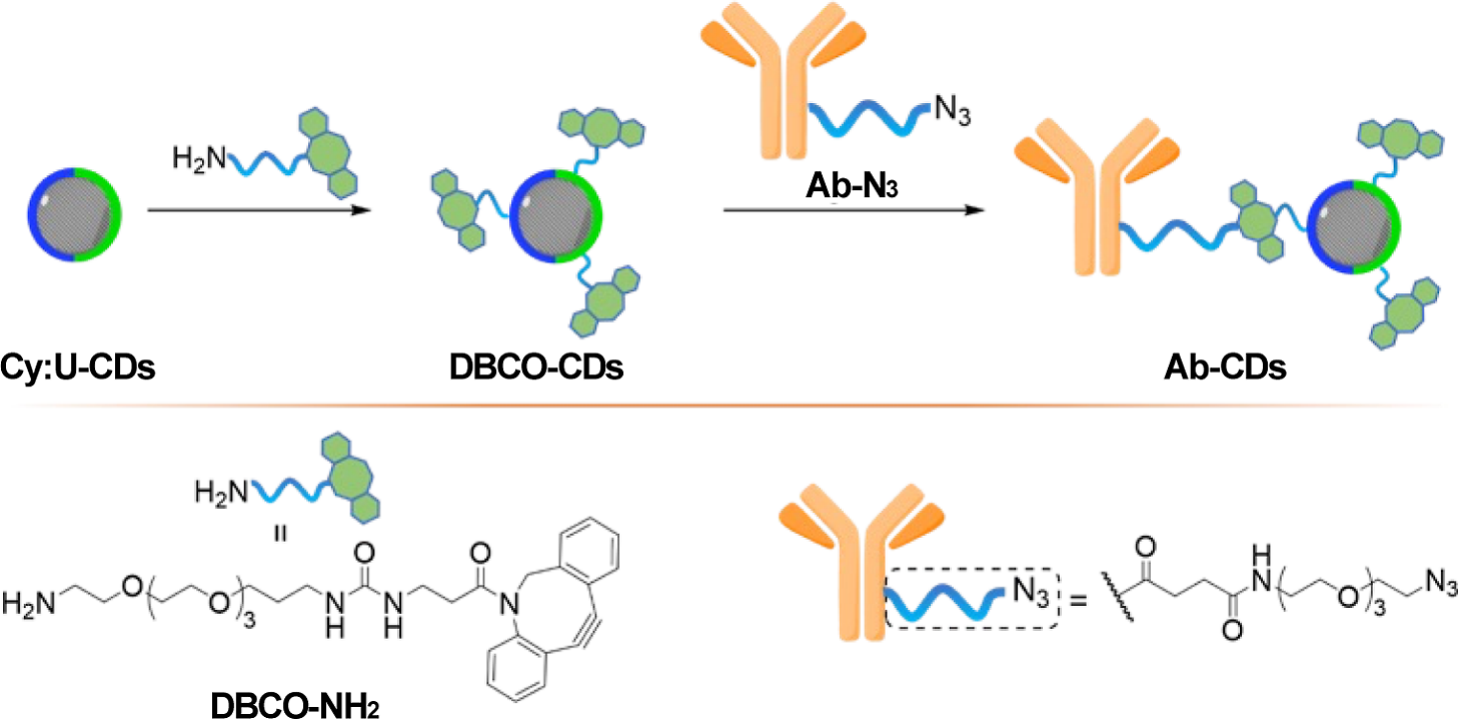
General Synthetic Ab-Conjugation Strategy Onto **Cy:U-CDs**

The surface of acid-coated **Cy:U-CDs** was successfully functionalized with dibenzocyclooctyl (DBCO) moieties using **DBCO-NH**_**2**_ in the presence of HATU, ready for facile conjugation to azide-functionalized antibodies (Abs) via the strain promoted azide–alkyne cycloaddition (SPAAC) reaction as previously described (Scheme 2, see Supporting Information for full details).^54^ The **DBCO-CDs** were then reacted with N_3_-modified anti-glial fibrillary acidic protein Abs (bearing 4 N_3_-linker moieties) to produce **Ab-CDs** adducts that could be used for the detection of glial fibrillary acidic protein, a tumor marker present in brain tissues of patients affected with glioblastomas (WHO grade 4 tumor) via immunostaining applications. Gel electrophoresis was used to confirm Ab-conjugation onto the CDs (Figure S29 in Supporting Information).

**Figure 5:**
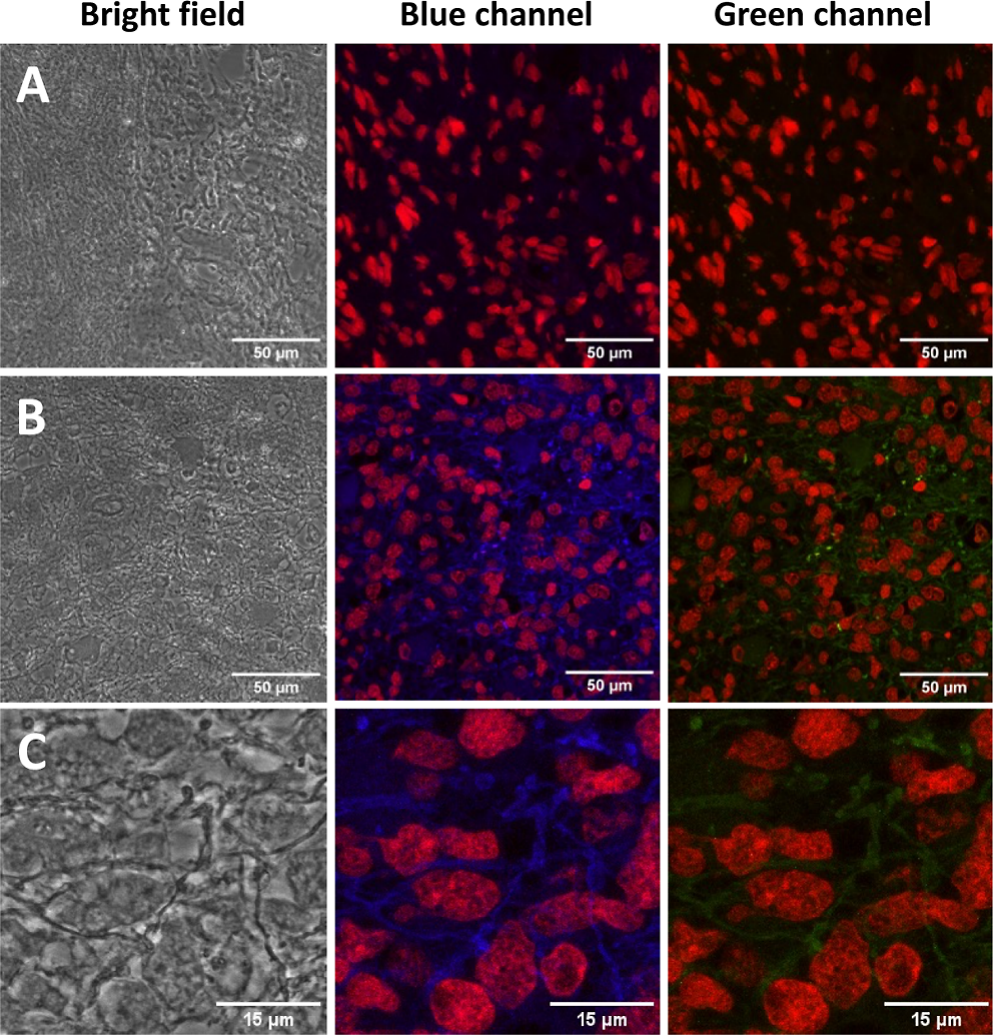
Confocal microscopy images of brain cancer tumor cells stained with Anti-GFAP **Ab-CDs**. Bright field and under 405 nm laser tracking the emission maxima for blue and green channels. The negative control schwannoma (a), showing immunofluorescence red labeling nuclei and negative for both blue and green GFAP staining (patient ID: 21/N1214A1). Immunostained tissue section of malignant brain tumor glioblastoma (b,c) stained with **Ab-CDs** showing immunofluorescence red labeling nuclei and blue/green labeling cobweb pattern of intermediate filament GFAP (patient ID: 08/0057B).

The presence of GFAP in 3 formalin-fixed paraffin-embedded biopsy brain tumor samples from different patients was then examined (2 glioblastomas, IDH wildtype, WHO Grade 4, and 1 negative control schwannoma, WHO Grade I, see Supporting Information Table S6) using the conjugated antibody **Ab-CDs**. Confocal analysis of stained tissues identified immunofluorescence within all the GBM cases (as assessed by a consultant neuropathologist KMK) using the **Ab-CDs** labeling of GFAP intermediate filaments to the GBM cell cytoplasm in both blue and green channels (Figures 5b,c, S27 and S28). The correct pattern of cytoplasmic staining (blue) of the GFAP intermediate filament in the GBM cell cytoplasm was identified. The intensity and extent of GFAP immunopositivity showed inter- and intra-tumoral heterogeneity in keeping with known biological variation between cases. Conversely, the immunostaining of schwannoma tissues, a tumor that does not express GFAP and thus was used as a negative control, showed no staining in both the blue and green detection channels as expected (Figure 5a). These results demonstrate the versatility of these novel probes in a clinical biomedical application. Further analysis of fluorescence intensities of blue and green channels showed a ratiometric value of 2.7 (blue/green) after background fluorescence subtraction for **Ab-CDs** labeled tissues of GBM patients, while the untreated cells showed a blue/green ratio of 3.5, which further confirms that the **Ab-CDs** probes are responsible for the labeling. Moreover, an increase of about 42% in fluorescence intensity for **Ab-CDs** labeled tissue was observed when compared to untreated or negative control tissues.

## Conclusions

The controlled synthesis of CDs with novel features offers unique opportunities for the design of fluorescent nano-scaffolds that are relevant to numerous applications. Multiple factors have been reported to alter the physico-chemical properties of these carbon-based materials. While there is a clear correlation between morphological/structural features and fluorescence modulation, the defined parameters that control the synthesis of tailored materials are still under debate. In this report, we describe a practical and reproducible strategy to access CDs with FQYs as high as 0.67 from simple commercial starting materials such as CA and Cy. We also demonstrate that the fluorescence of the CDs can be tuned by modifying the synthetic protocols using heteroatom-enriched co-precursor to yield CDs with multiple PLCs. The bright blue emission in **Cy-CDs** was found to correlate to the presence of molecular TPA, while modifying the reaction pathways to generate other N-containing aromatic motifs results in excitation-dependent behaviors with materials exhibiting dual emission profiles. We rationalize this is due to the coexistence of noninteracting PLCs and the heterogeneity of the hybridized structure which leads to the unique optical properties of these materials. Moreover, multicolor fluorescent bands with excitation-independent behaviors in co-doped CDs suggest that isolated fluorophores are being formed and trapped within the CDs structure. Structural analysis using AFM confirmed the formation of nanostructures, while NMR, FTIR, and XPS demonstrated each material features different surface functionalities and graphitic groups that are believed to regulate the emission spectra. In this manner, the green fluorescence in **Cy:U-CDs** can be attributed to amine and pyrrolic functional groups, whereas for **Cy:EG-CDs**, the molecular states that give its yellow emission correlate to C–OH/C–O–C motifs. On the other hand, it was found that the composition of **Cy:F-CDs** largely contributes to a shift of the fluorescence to longer wavelengths. Thus, it is possible that its red emission originates from a high content of imide N within the core. Further, the utility of these new classes of materials was successfully utilized in a bioimaging application with the **Cy:U-CDs** conjugation with anti-GFAP Abs for immunostaining of human brain tumor tissues clinical samples, demonstrating the versatility of these novel dual fluorescent nanoprobes.

## Materials and Methods

### Synthesis of Cy-CDs and Cy:U-CDs

Citric acid (CA) (1.00 g, 5.2 mmol) was dissolved in distilled H_2_O (20 mL) in a 250 mL conical flask. l-cysteine (Cy) (0.787 g, 6.50 mmol) was then added to the solution. In the case of **Cy:U-CDs**, urea (U) (4.92 g, 83.11 mmol) was added to the solution. Then, the mixture was stirred for 20 min to ensure homogeneity. The conical flask was then placed in a domestic microwave at 600 W (inside a fume cupboard) and the solution was reacted for 3 min. A viscous amber residue was obtained which was washed with acetone (4 times). The precipitate was then phase-separated by centrifugation and re-dissolved in 15 mL of distilled H_2_O. The CD solution was purified via centrifuge-filtration using Vivaspin concentrators (MWCO 10 kDa, 8500 rpm, 30 min). The filtrate solution was concentrated in vacuo to yield an amber powder of **Cy-CDs** (1.22 g) and **Cy:U-CDs** (1.05 g), respectively.

### Synthesis of Cy:EG-CDs

CA (0.3 g, 1.56 mmol) was dissolved in ethylene glycol (EG) (30 mL, ρ 1.11 g/mL). Then, Cy (0.94 g, 7.8 mmol) was added to the solution and stirred for 20 min to ensure homogeneity. The solution was then transferred to a Teflon autoclave reactor and heated at 180 °C for 3 h. A viscous amber solution was obtained which was then concentrated under reduced pressure and redissolved in 10 mL of distilled H_2_O. The residue was then washed with Et_2_O (4 times) and the aqueous layer was phase-separated. The CD solution was purified via centrifuge-filtration using Vivaspin concentrators (MWCO 10 kDa, 8500 rpm, 30 min). The filtrate solution was lyophilized to yield an amber powder of **Cy:EG-CDs** (0.19 g).

### Synthesis of Cy:F-CDs

CA (0.3 g, 1.56 mmol) was dissolved in formamide (F) (30 mL, ρ 1.13 g/mL). Then, Cy (0.94 g, 7.8 mmol) was added to the solution and stirred for 20 min to ensure homogeneity. The solution was then transferred to a Teflon autoclave reactor and heated at 180 °C for 3 h. A dark red solution was obtained which was then concentrated under reduced pressure and redissolved in 10 mL of distilled H_2_O. The residue was washed with acetone (4 times), then, the precipitate was phase-separated by centrifugation and re-dissolved in 15 mL of distilled H_2_O and the aqueous phase-separated The CD solution was purified via centrifuge-filtration using Vivaspin concentrators (MWCO 10 kDa, 8500 rpm, 30 min). The filtrate solution was lyophilized to yield an amber powder of **Cy:F-CDs** (0.24 g).

### Synthesis of DBCO-CDs

To a stirred solution of **Cy:U-CDs** (9.4 mg) in dry DMF (1 mL), HATU (41 mg, 0.11 mmol) and DIPEA (19 μL, 0.11 mmol) were added and the solution was allowed to stir for further 15 min at room temperature. A solution of **DBCO-NH**_**2**_(54) (28.2 mg, 0.05 mmol) was added to dry DMF (0.5 mL) and the solution was stirred at room temperature for 5 h. H_2_O (0.5 mL) was then added to quench the reaction and the solution was stirred for further 10 min at room temperature and concentrated under reduced pressure. The residue was redissolved in aq. NaOH solution (1.5 mL, 0.1 M solution) and stirred for 1 h at room temperature. The pH was neutralized with the addition of aq. HCl solution (0.15 mL, 1 M solution), diluted with H_2_O (20 mL), and washed with Et_2_O (5 × 10 mL), and the water phase was concentrated under reduced pressure. The residue was purified via 0.5–1 KDa cutoff dialysis membrane against water, changing the water 3 times over a 24 h period. The dialyzed solution was then freeze-dried furnishing **DBCO-CDs** (9.9 mg) as a dark solid. Note: The FQY of DBCO-CDs upon **DBCO-NH**_**2**_ conjugation was reduced (Figure S28) with FQY values of 0.070 (for blue emission at 450 nm) and 0.082 (for green emission at 499 nm) with no shift on fluorescence emission when compared to the unconjugated Cy:U-CDs (FQY 0.15 and 0.219).

### Ab-CDs Conjugation Procedure

Commercial GFAP antibodies were functionalized with different amounts of **N**_**3**_**-PEG-NHS** linker^54^ (0.1 mg/μL in DMSO stock solution) 0.12, 0.24, and 1.20 μmol corresponding to about 108, 217, and 1086 equiv respectively furnishing three different Ab-N_3_**S1-3** with approx. 4.41, 8.57, and 26.36 N_3_ moieties per antibody respectively. Compounds **S1-3** were conjugated via SPAAC reaction by simple mixing with **DBCO-CDs** furnishing **Ab-CDs S4–S6**. The effectiveness of the conjugation reaction was verified via gel electrophoresis analysis of Ab-CD adducts **S4-6** (Figure S29). Compared with native unfunctionalized Abs (Figure S29, i) CD-functionalized Abs showed an increased molecular weight correlating with the increased amount of **DBCO-CDs** moieties present on the Ab surface (Figure S29 ii–iv).

### Tissue Staining

Brain tissue samples were stained following the protocol previously described by our group and analyzed with confocal microscopy acquiring the fluorescence emission in both the blue and green fluorescence region of **Ab-CDs S4**. The clinical data for the samples scanned are included in Table S6 (Supporting Information). In brief, the tissue sections were deparaffinized and rehydrated as follows: the sections were incubated in three washes of xylene for 2 min each, followed by two washes of 100%, 95% ethanol for 10 min each. The sections were then washed twice in distilled H_2_O for 5 min each. The tissue slides were then placed in the microwaveable vessel. Tris–EDTA antigen retrieval buffer (10 mM Tris base, 1 mM EDTA solution, 0.05% Tween 20, pH 9.0) was added and placed inside the microwave, which was set to full power until the solution came to a boil. The solution was boiled for 20 min from this point and left on the bench at room temp to cool for 30 min. The slides were then washed for 2 × 5 min with TBS plus 0.025% Triton X-100 with gentle agitation. The slides were blocked in Superblock buffer (Thermofisher, ref 37,515) for 30 min at room temp. The slides were drained for a few seconds (not rinsed) and wiped around the sections with tissue paper. 400 μL of CDs-conjugated GFAP antibody (1:500) was then added per slide and incubated at 4 °C overnight. The slides were then rinsed 3 × 5 min with TBS plus 0.05% Tween20.

Nuclear stain: The slides were equilibrated with 300 μL buffer 2xSSC (0.3 M NaCl, 0.03 M sodium citrate, pH = 7.0) 2 × 3 min, then 150 μL (500 nM) propidium iodide/PI were added per slide, incubated at 37 °C incubator for 5 min. Afterward, the slides were washed 6 times with buffer 2xSSC 300 μL. The slides were mounted using mounting medium fluoromount-G and a coverslip was added. Clear nail polish was added to seal the edges around the coverslip.

## Supporting Information Available

Full experimental details and structural characterization and fluorescence studies of CDs, antibody conjugate preparation, and tissue staining (PDF)
