# Supplementary material for: Multicolor Photoluminescent Carbon Dots à La Carte for Biomedical Applications
Source: ACS Appl Mater Interfaces. 2023 Sep 16;15(38):44711–21. doi: 10.1021/acsami.3c08200 (PMC10540137; doi:10.1021/acsami.3c08200)
Supplement: Supplementary file 1 — am3c08200_si_001.pdf [file am3c08200_si_001.pdf]

SUPPORTING INFORMATION

Multicolour dual-emission Photoluminescent Carbon Dots à la carte for biomedical applications

Teodoro Garcia-Millan,[a] Javier Ramos-Soriano,[a] Mattia Ghirardello,[a] Xia Liu,[d] Cristina Manuela Santi, [a] Jean Charles Eloi, [a] Natalie Pridmore, [a] Robert L. Harniman,[a] David J. Morgan,[b,e] Stephen Hughes,[c] Sean A. Davis,[a] Thomas A.A. Oliver,[a] Kathreena M. Kurian*,[d] and M. Carmen Galan*[a]*

[a] School of Chemistry, University of Bristol, Cantock's Close, Bristol, BS8 1TS, UK

[b] Cardiff Catalysis Institute, Cardiff University, Park Place, Cardiff, CF10 3AT, UK and HarwellXPS – The EPSRC National Facility for Photoelectron. Spectroscopy, Research Complex at Harwell (RCaH), Didcot, OX11 0FA, UK

[c] DST Innovations Ltd, Unit 6a Bridgend Business Centre, Bennett Street, Bridgend, CF31 3SH, UK

[d] Bristol Medical School, Public Health Sciences, Southmead Hospital, University of Bristol, Southmead Road, Bristol, BS8 NB, UK.

* Corresponding Authors

1. General

Reagents and solvents were purchased as reagent grade from Sigma Aldrich or Fisher and used without further purification. CDs synthesis was conducted in either a domestic microwave (Wilko's Homebrand) or a Teflon-lined autoclave. Concentration centrifugation tubes were GE Healthcare Life Sciences VIVASPIN 6 or 20, with a 10 kDa MWCO filter. Biotech-Grade CE Dialysis Tubing, 500-1000 MWCO, 31mm/20mm; 33ft was purchased from Cole-Parmer. Extracts were concentrated under reduced pressure using both a Büchi rotary evaporator at a pressure of 0.1 mm Hg (oil pump), as appropriate, and a high vacuum line at room temperature. ^{1}H (Presaturation), ^{13}C (HSQC) and Diffusion-Ordered (DOSY) NMR spectra were measured in D_2O and DMSO-d_6 at 500 MHz on a Varian spectroscope, their chemical shifts are quoted in parts per million (ppm) and referenced to the residual solvent peak (D_2O: ^{1}H = 4.79 ppm). Atomic force microscopy (AFM) was carried using a multi-mode VIII microscope with Nanoscope V control utilising a Fast-scan head unit whilst operating under PeakForce feedback control, using a SCANASYST-AIR-HR cantilever (Bruker, CA, USA) with a nominal spring constant of 0.4 N/m. Tip deconvolution using a Ti roughness sample (RS-12M; Bruker, CA, USA) revealed a tip radius of 4.98 nm from the tip apex. X-ray photoelectron spectroscopy (XPS) was performed using a Kratos Axis Ultra DLD system, using a monochromatic Al *Kα* X-ray source operating at 140 W power (10 mA x 14 kV). Data was collected with pass energies of 160 eV for survey spectra, and 20 eV for the high-resolution scans with step sizes of 1 eV and 0.1 eV, respectively. Samples were either pressed onto doubled sided Scotch tape (type 665) or for viscous samples, spread onto a UV cleaned Si wafer. The system was operated in the Hybrid mode, using a combination of magnetic immersion and electrostatic lenses, and acquired over an area of approximately 300x700 μm^2. A magnetically confined charge compensation system was used to minimize charging of the sample surface, and all spectra were taken with a 90° take of angle. A base pressure of ~ 1×10^{-9} Torr was maintained during the collection of the spectra. Data were analysed using CasaXPS (v2.3.23) after subtraction of a Shirley background and using modified Wagner sensitivity factors as supplied by the manufacturer. Absorbance measurements were conducted in H_2O on a Cary UV-Vis 50 spectrophotometer in 3500 μL quartz cuvettes (ThorLabs). Fluorescence measurements were obtained in H_2O with a Perkin-Elmer LS-45 and samples were diluted (adjusted to an absorption lower than 0.1 at excitation wavelength) prior to analysis to avoid effects of self-absorption on the emission spectra. Quantum yield (QY) of fluorescence measurements were conducted between both Perkin-Elmer LS-45 and Cary UV-Vis 50 spectrophotometers in quartz cuvettes based on IUPAC protocol. [1] The calculous was estimated relative to quinine sulphate (QY = 0.6, 0.1 M H_2SO_4), coumarin 153 (QY = 38%, EtOH) or tris(bipyridine)ruthenium(II) chloride (QY = 0.063, H_2O), respectively. For the design of fluorescent biotargets, Polyclonal Rabbit Anti-Glial Fibrillary Acidic Protein (Concentrate) was purchased from Agilent Dako (Z033401-2). Gel electrophoresis was carried out on NuPAGE 4-12% Bis-Tris Gel purchased from Invitrogen in MES buffer using a Bio-Rad 1000/500 electrophoresis power supply. Proteins were loaded at a similar concentration and stained with PageBlue™ protein staining solution. Paraffin fixed slides of brain tissue from different patients were kindly provided by the Southmead Hospital, University of Bristol, Bristol, United Kingdom. Cells were stained using propidium iodide purchased from Thermofisher (P3566) for the nuclei, and compounds **S4-S6** for GFAP. Confocal microscope

images were acquired on a Leica DMi8 inverted epifluorescence microscope using the blue lines of the argon-ion laser (405 nm) and tuneable white light lasers and 63x (NA 1.4) objective at the Wolfson Imaging facility at the University of Bristol. The images were analysed using Fiji (ImageJ) software.

2. Reaction monitoring of Cy-CDs

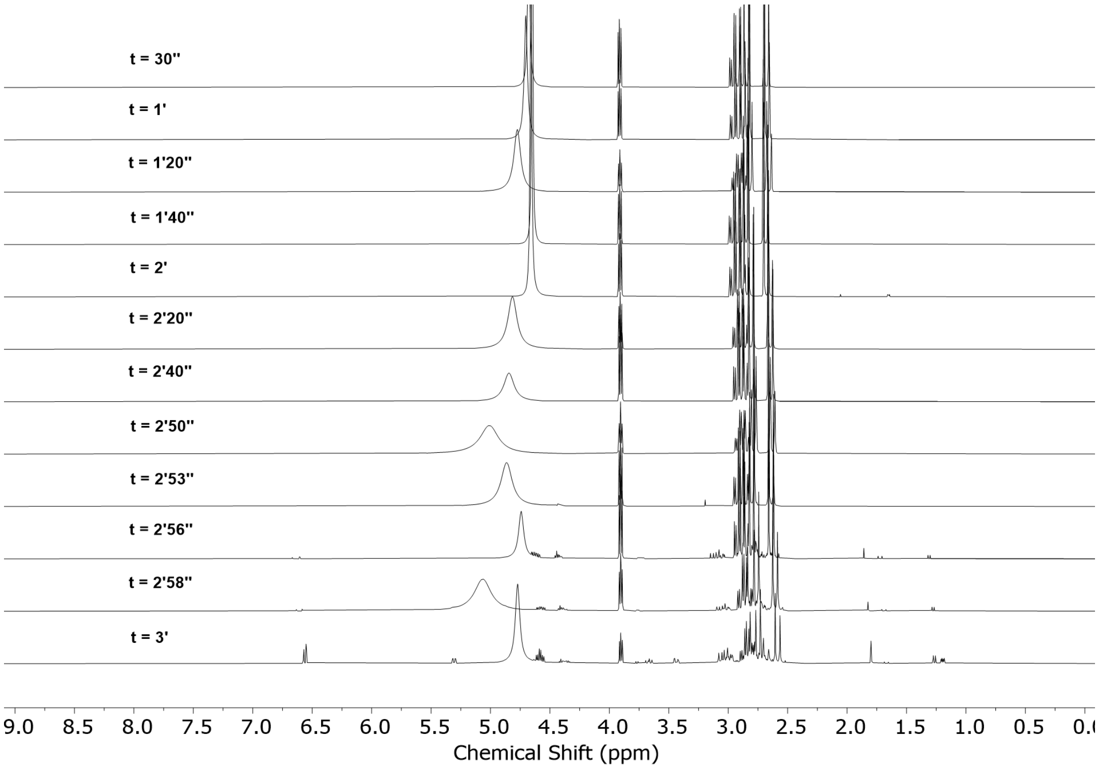

Figure S1. Series of ^{1}H PRESAT NMR spectra of raw samples in **Cy-CDs** synthesis at different reaction times (D_2O, 500 MHz).

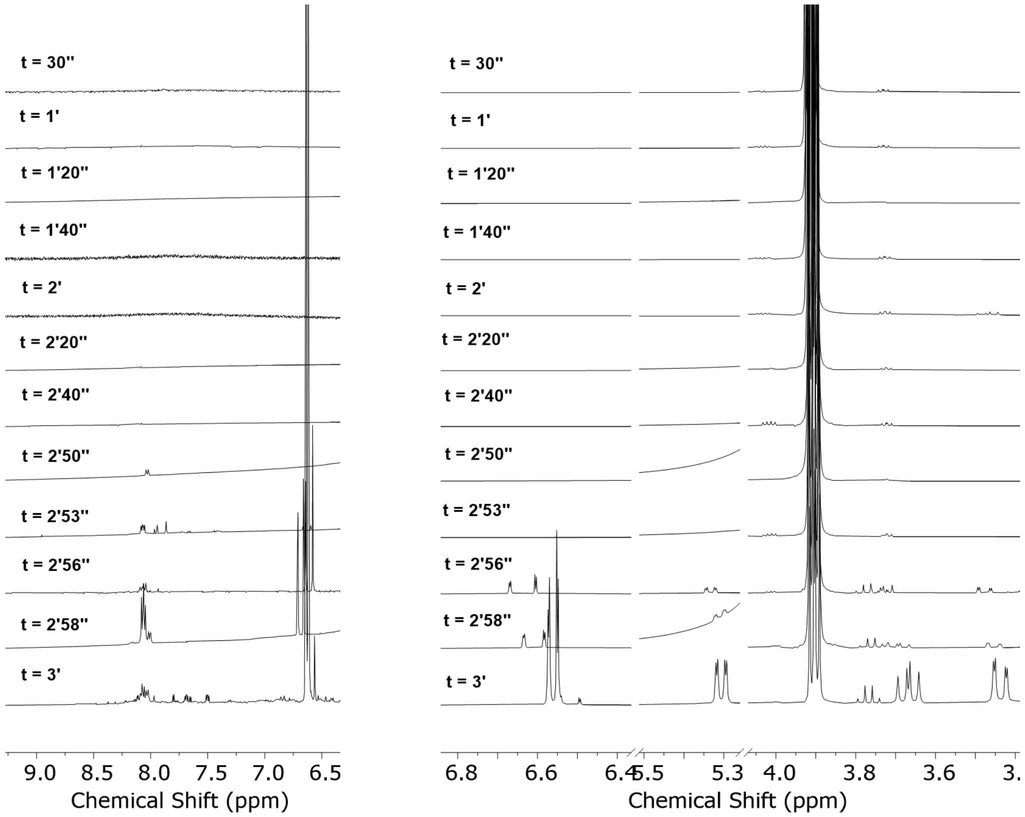

Figure S2. Series of ^{1}H PRESAT NMR spectra of raw samples in **Cy-CDs** synthesis at different reaction times (D_2O, 500 MHz).

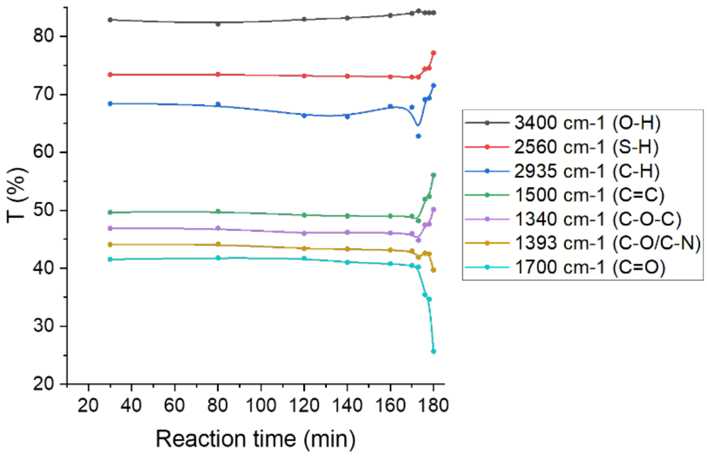

Figure S3. Transmission percentage of several wavenumbers at different reaction times in the **Cy-CDs** synthesis. Data extracted from IR spectra of raw samples series.

3. Fluorophore in Cy-CDs

5-oxo-2,3-dihydro-5*H*-(1,3)thiazolo(3,2-a)pyridine-3,7-dicarboxylic acid (TPA)

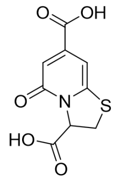

The synthesis of 5-oxo-2,3-dihydro-5*H*-(1,3)thiazolo[3,2-*a*]pyridine-3,7-dicarboxylic acid (TPA) was an adaptation the protocol reported by Wang *et al.* [2] 5 g of CA and 2.85 g of Cys were added to 5 mL of H_2O in a 10 mL round flask. The solution was stirred for 15 min and heated during 72 hours keeping reflux set up. The resulting gel was dissolved in 15 mL H_2O and the product was recrystallised from H_2O to give a light yellow solid. **^{1}H NMR** (500 MHz, D_2O) characteristic signals, δ 6.79 (d, J = 1.53 Hz, 1H, 3-pyridine), 6.77 (d, J = 1.56 Hz, 1H, 5-pyridine), 5.57 (dd, J = 9.01, 2.01 Hz, 1H, -C*H*-COOH), 3.9 – 3.67 (m, 2H, -C*H*$_2$-S-). **^{13}C NMR** (126 MHz, D_2O) δ 171.82 (C-1), 167.84 (C-2), 163.53 (C-3), 150.96 (C-4), 144.29 (C-5), 114.64 (C-6), 101.7 (C-7), 64.67 (C-8), 31.87 (C-9).

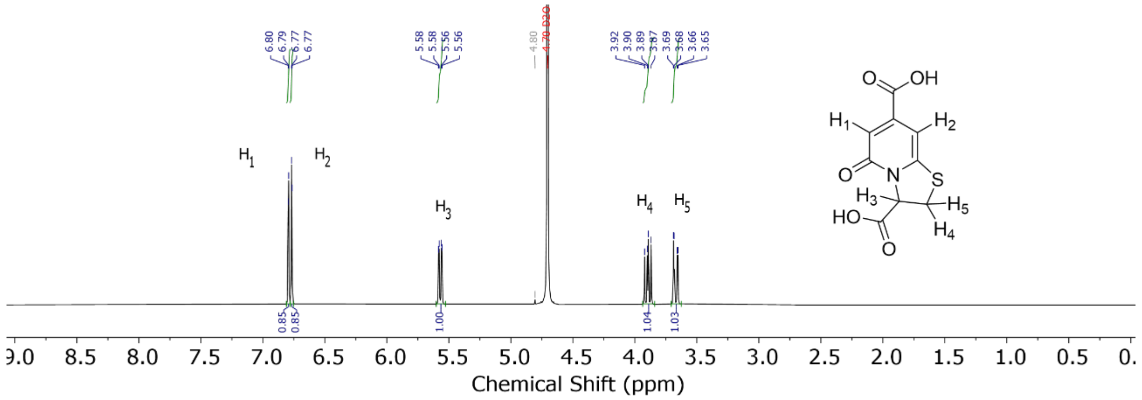

Figure S4. ^{1}H PRESAT NMR spectrum of TPA (D_2O, 500 MHz).

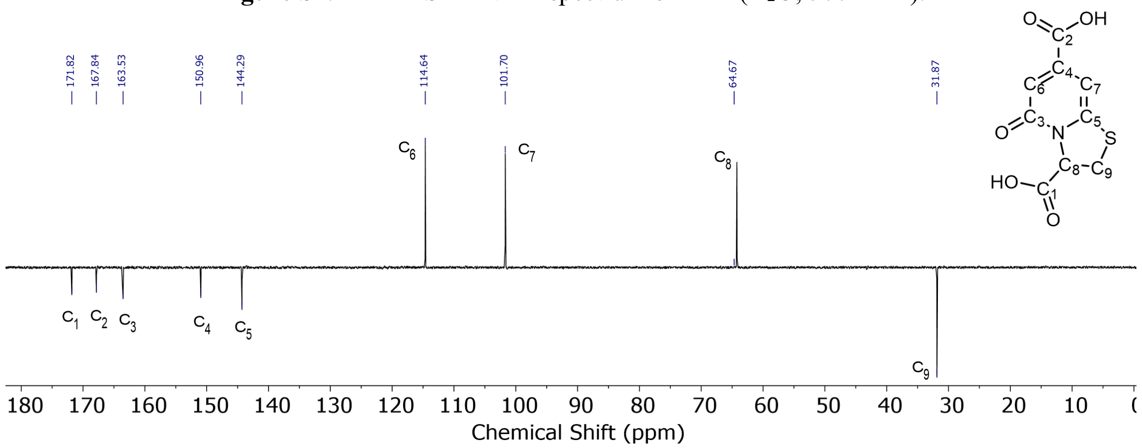

Figure S5. ^{13}C APT NMR spectrum of TPA (D_2O, 500 MHz).

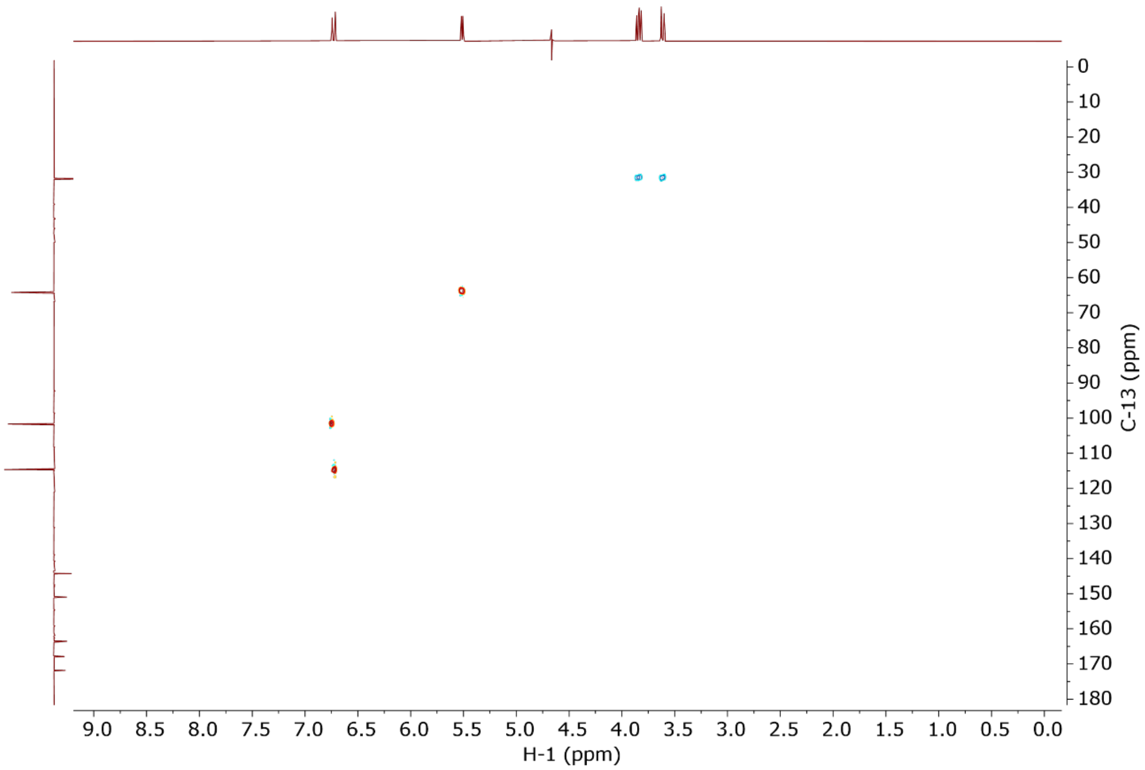

Figure S6. ^{1}H-^{13}C HSQC NMR spectrum of TPA (D_2O, 500 MHz).

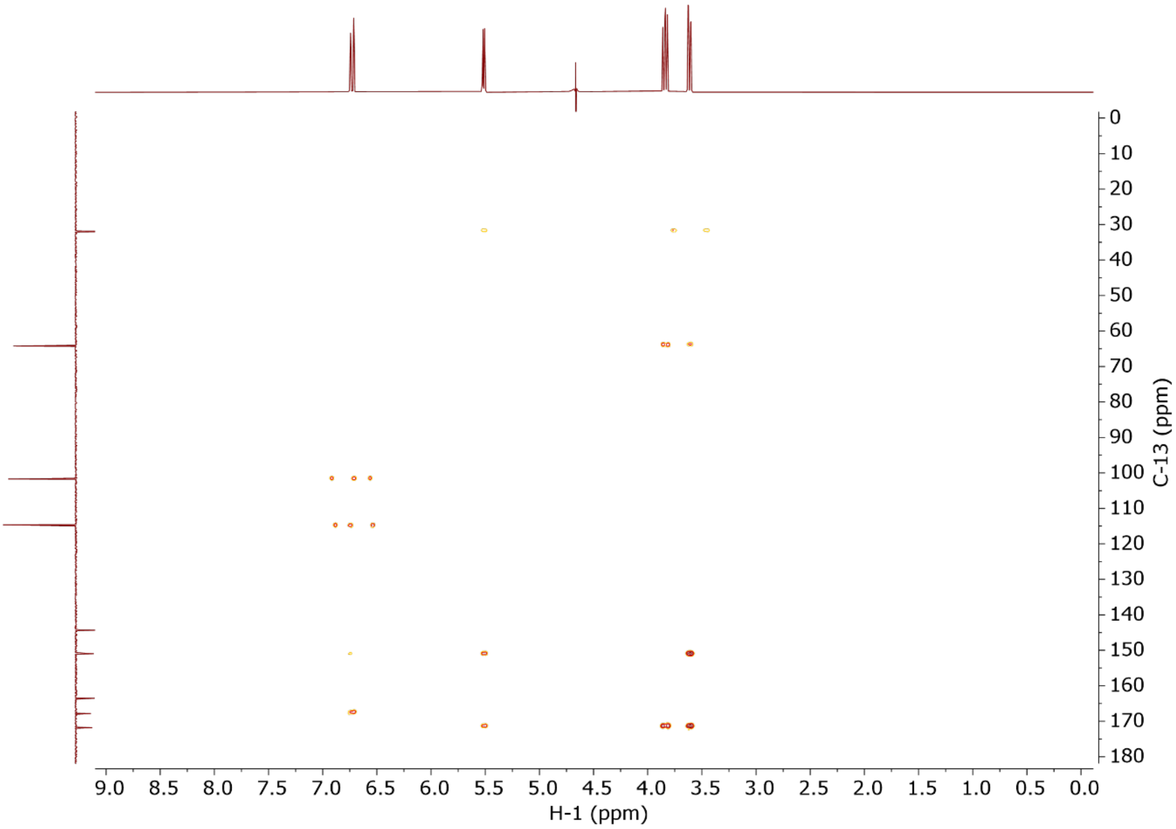

Figure S7. ^{1}H-^{13}C HMBC NMR spectrum of TPA (D_2O, 500 MHz).

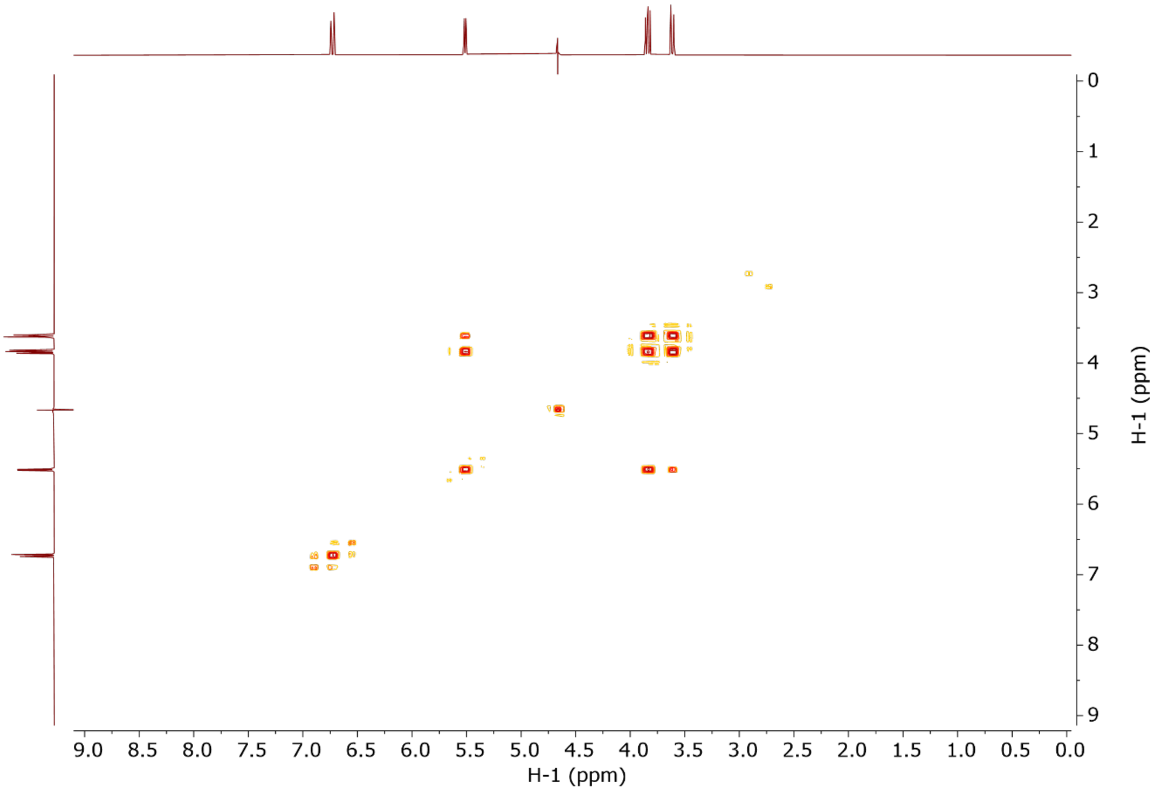

Figure S8. ^1H-^1H COSY NMR spectrum of TPA (D_2O, 500 MHz).

TPA identification

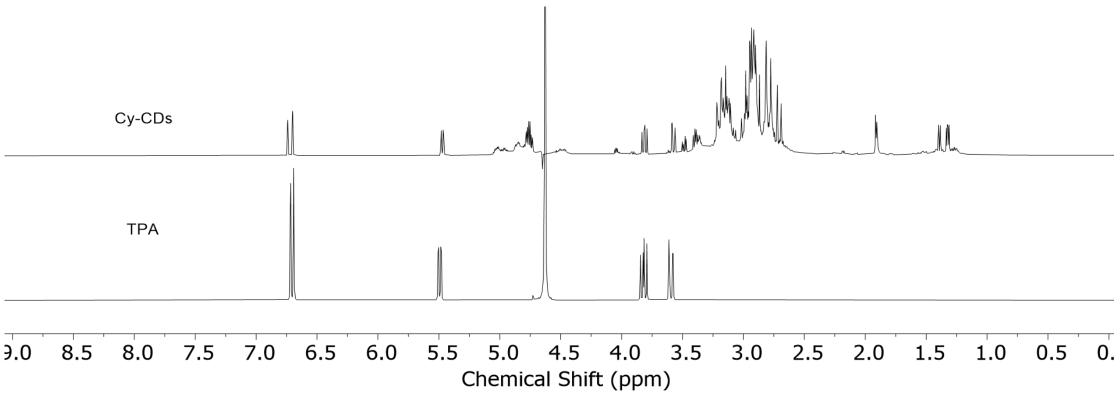

Figure S9. **Cy-CDs** and TPA ^1H PRESAT NMR spectra correlation (D_2O, 500 MHz).

A)

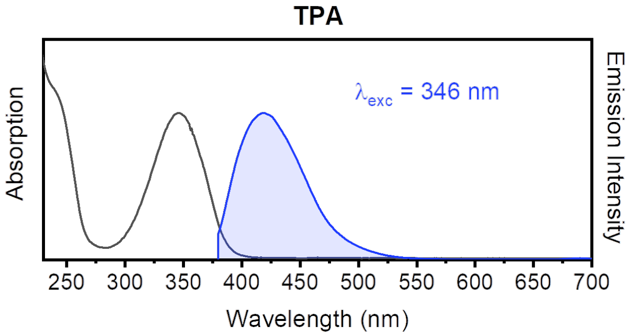

B)

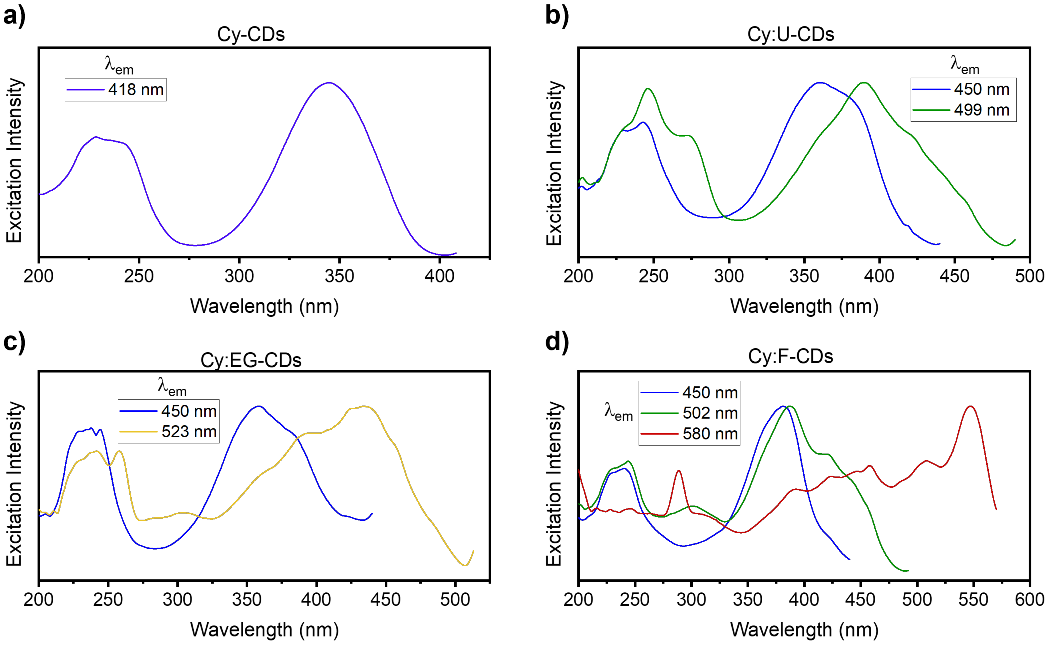

Figure S10. A) UV-Vis absorption and emission spectra of TPA. **B)** Excitation spectra of CDs for different emission wavelengths: **Cy-CDs** (a), **Cy:U-CDs** (b), **Cy:EG-CDs** (c) and **Cy:F-CDs** (d).

4. X-Ray Powder diffraction (XRD)

For the powder XRD measurements a Bruker D8 Advance with a PSD LynxEye detector and Cu radiation (1.540600 Angstrom) was used. Materials were measured between 2θ = 5 and 50°, with a step size of 0.02° and an exposure of 1 s/step.

Samples **Cy:F-CDs** (c) and **Cy:EG-CDs** (d) displayed crystalline peaks which did not correspond to the starting materials L-cysteine and citric acid (Figure S11).

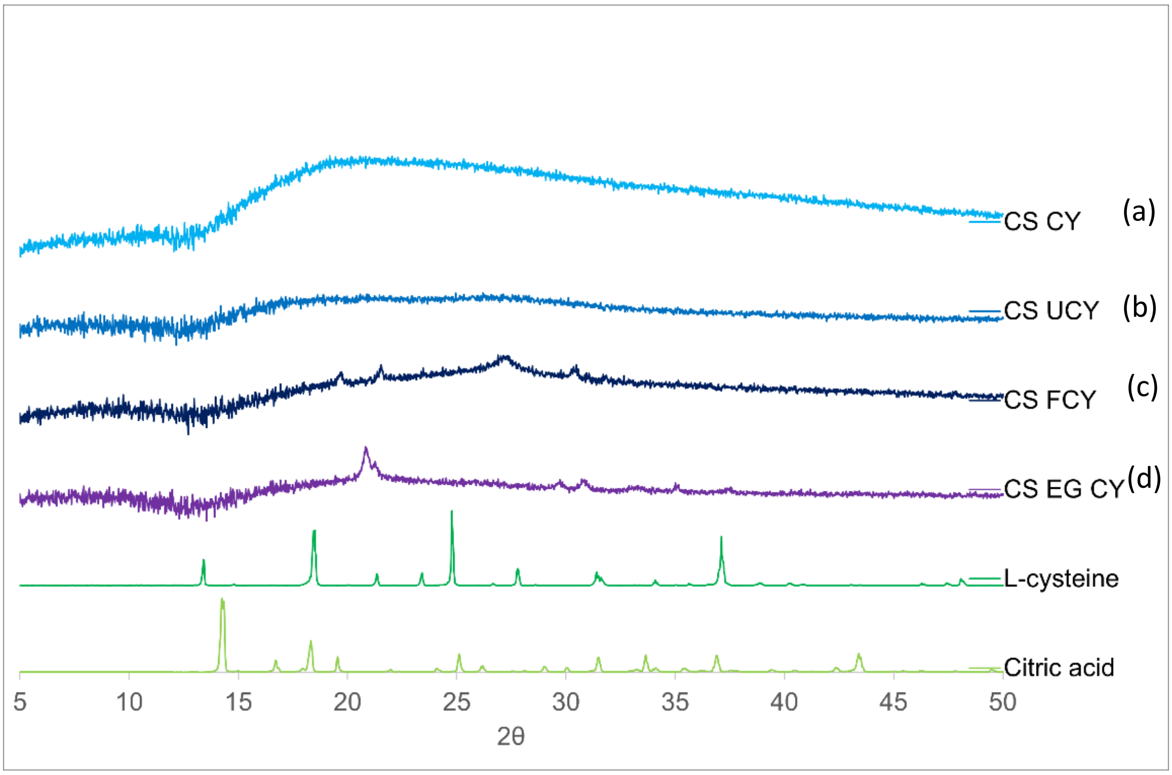

Figure S11. Powder X-ray diffraction spectra from the four carbon dot samples (**Cy-CDs** (a), **Cy:U-CDs** (b), **Cy:EG-CDs** (c) and **Cy:F-CDs** (d) and starting materials (L-cysteine and citric acid).

5. Transmission electron microscopy (TEM)

Transmission electron microscopy specimens were prepared from dispersions of the powder samples in EtOH (except CS-Cys where water was used). Sample dispersions were drop-cast (50 microlitre) onto 3 mm carbon-covered 200 mesh copper grids. Specimens were imaged on a field-emission gun JEM-2100F from JEOL, Japan, at 200kV, equipped with an Orius SC1000 camera from Gatan, US.

Carbon dots were observed by TEM studies of all four samples which were typically less than 10 nm in diameter (Figure S12). Samples **Cy:EG-CDs** and **Cy-CDs** were stable to high resolution imaging and interplanar spacings of ~2.55Å and ~2.21Å, were determined, respectively (Figure S13). It has not been possible to obtain interplanar spacings from samples **Cy:U-CDs** and **Cy:F-CDs** currently.

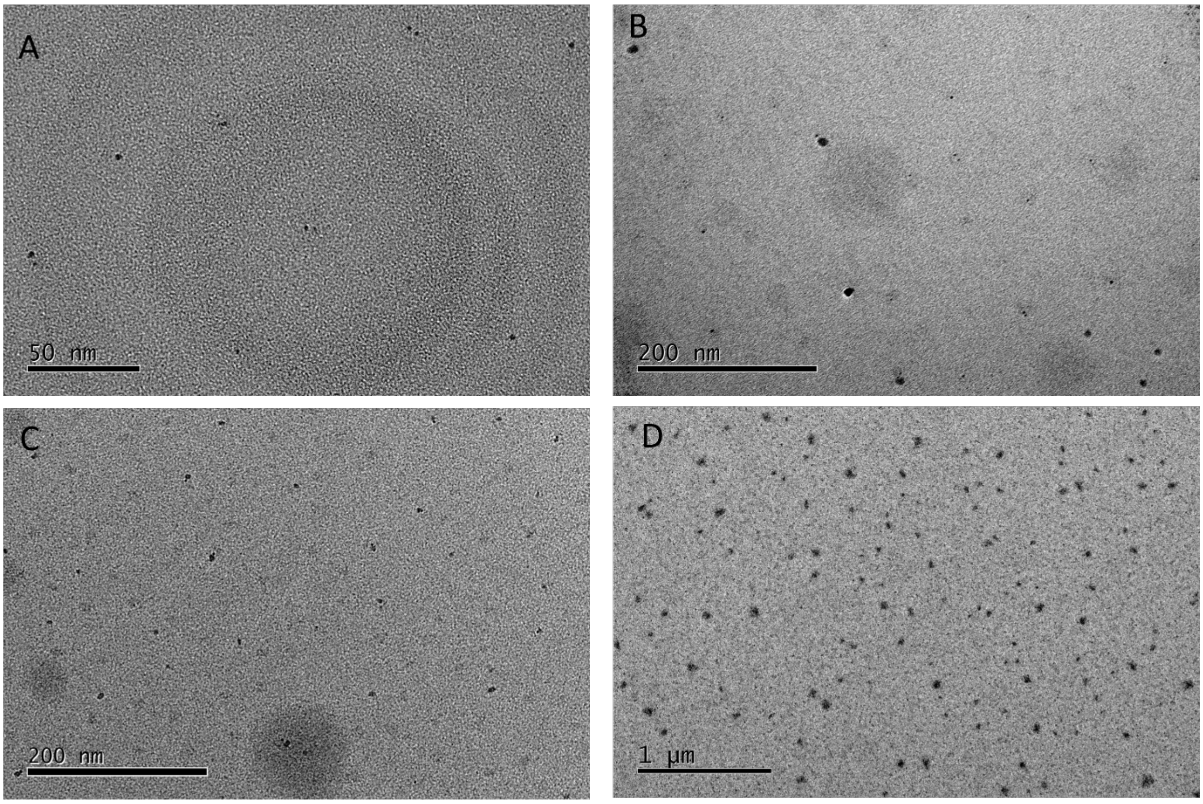

Figure S12. HRTEM images of **Cy-CDs** (A), **Cy:EG-CDs** (B), **Cy:U-CDs** (C), and **Cy:F-CDs** samples.

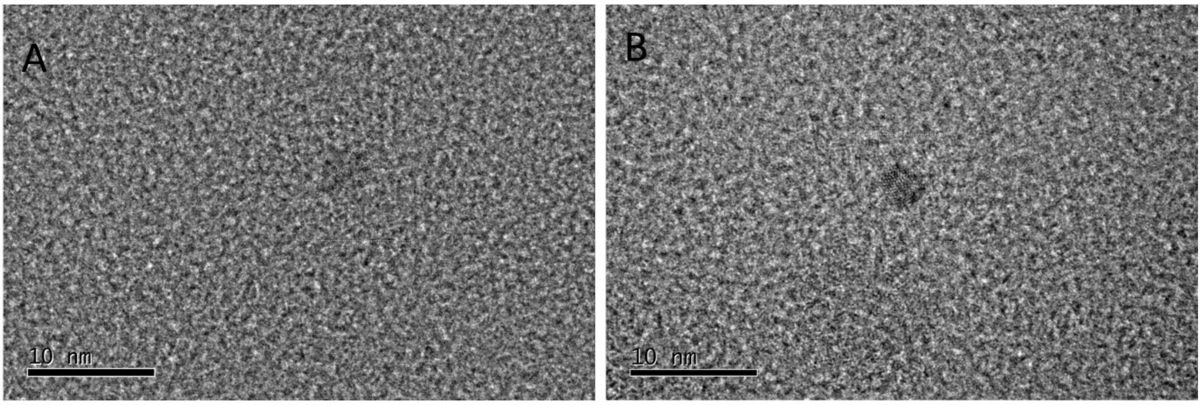

Figure S13. HRTEM images of **Cy-CDs** (A) and **Cy:EG-CDs** (B) samples.

6. Atomic Force Microscopy (AFM)

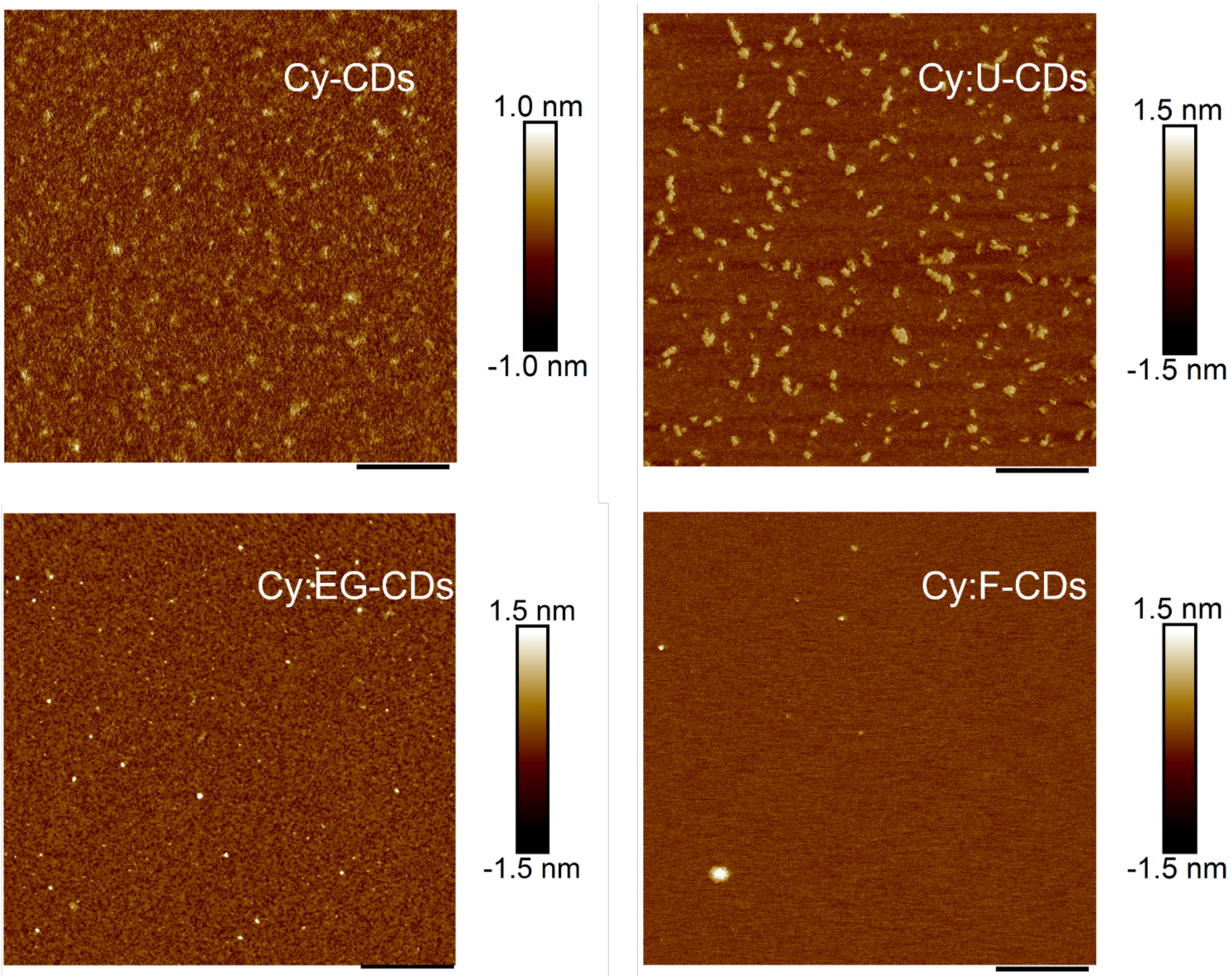

Figure S14. 2D AFM images of CDs. The black scale bar represents 800 nm.

7. NMR Spectra Co-doped CDs

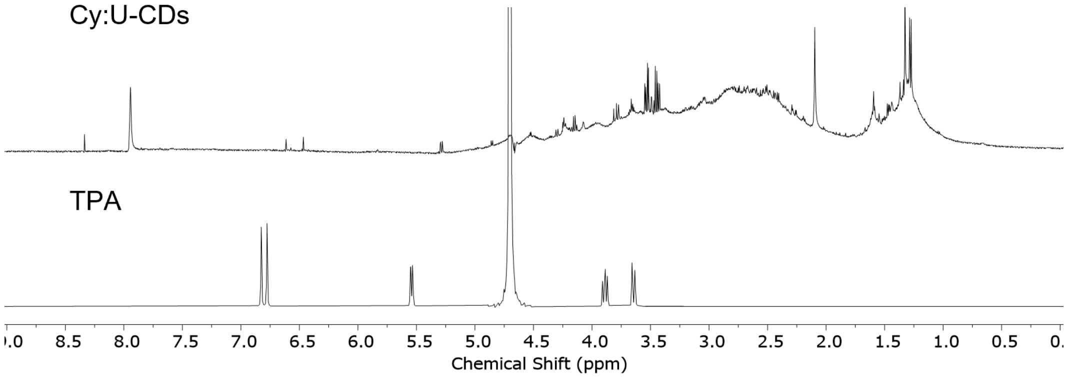

Figure S15. **Cy:U-CDs** and TPA ^{1}H PRESAT NMR spectra correlation (D_2O, 500 MHz).

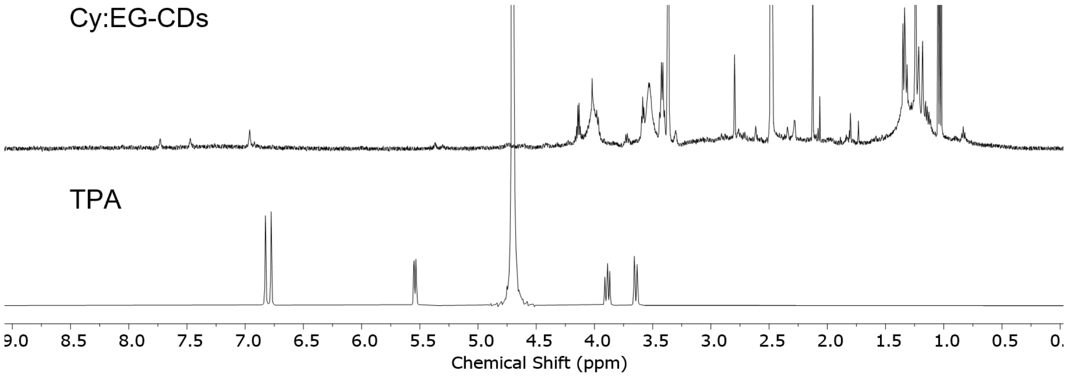

Figure S16. **Cy:EG-CDs** and TPA ^{1}H PRESAT NMR spectra correlation (DMSO-d6, 500 MHz).

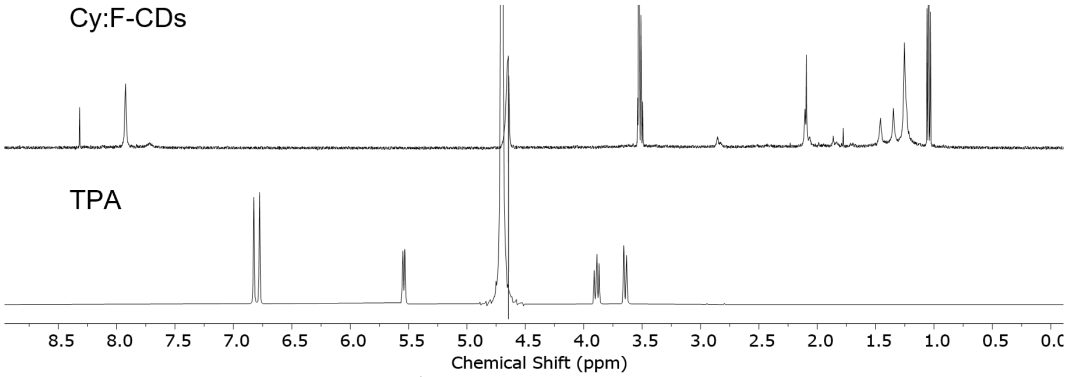

Figure S17. **Cy:F-CDs** and TPA ^{1}H PRESAT NMR spectra correlation (D_2O, 500 MHz).

8. Diffusion-Ordered NMR Spectroscopy (DOSY)

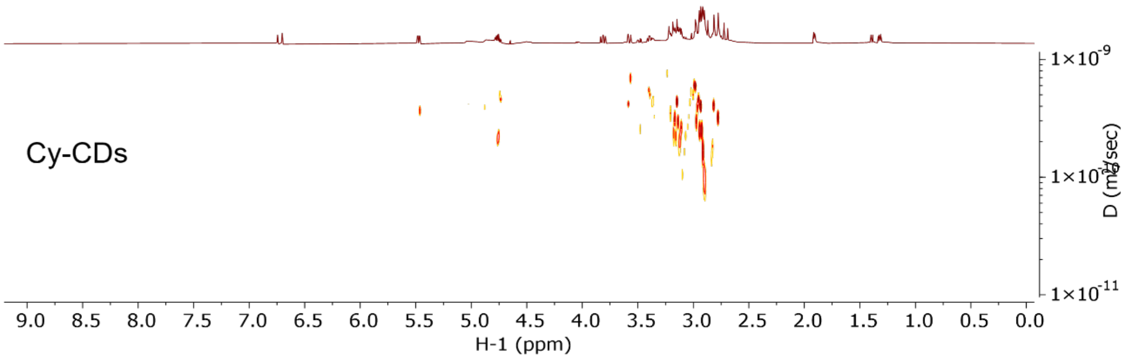

Figure S18. ^{1}H DOSY NMR of **Cy-CDs** (D_2O, 500 MHz).

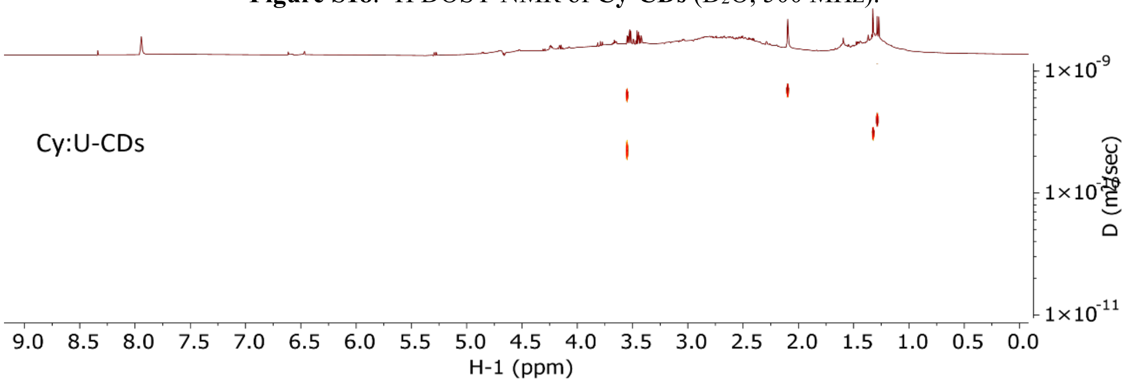

Figure S19. ^{1}H DOSY NMR of **Cy:U-CDs** (D_2O, 500 MHz).

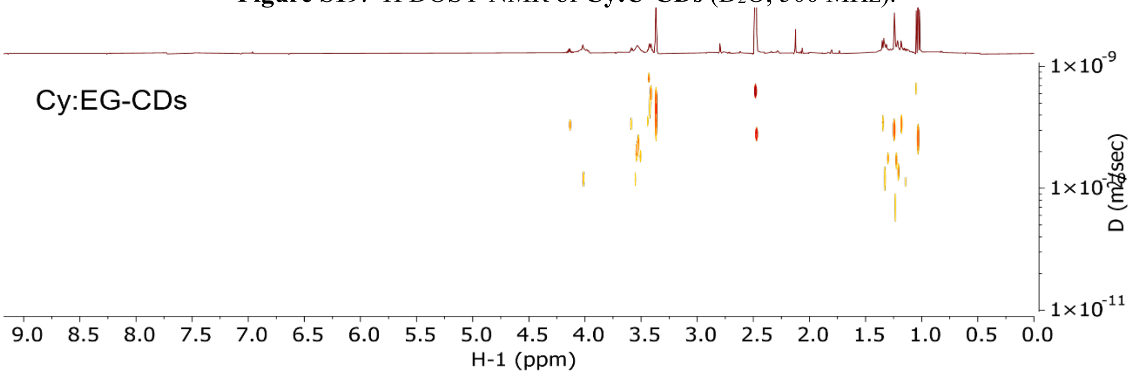

Figure S20. ^{1}H DOSY NMR of **Cy:EG-CDs** (D_2O, 500 MHz).

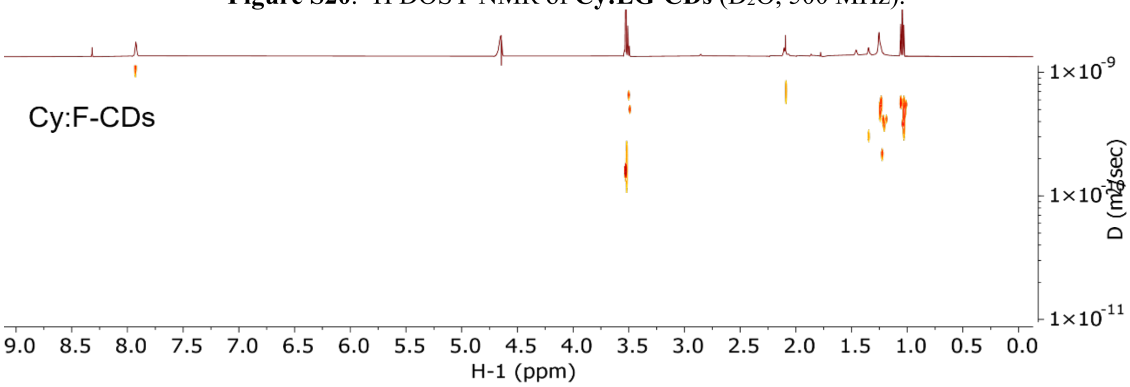

Figure S21. ^{1}H DOSY NMR of **Cy:F-CDs** (D_2O, 500 MHz).

9. Fluorescence Quantum Yield

Fluorescence	λ_{exc} (nm)	FQY (respect to standard)		
		Quinine Sulphate	Coumarine 153	$Ru(bpy)_3^{2+}$
Cy-CDs (A)	346	0.669		
Cy:U-CDs (A)	340	0.15		
Cy:U-CDs (B)	391		0.219	
Cy:EG-CDs (A)	369	0.103		
Cy:EG-CDs (B)	443		0.13	
Cy:F-CDs (A)	390	0.228		
Cy:F-CDs (B)	430		0.154	
Cy:F-CDs (C)	550			0.442

Table S1: FQY measurement of CDs specific to band A, B and C respect to standards: Quinine Sulphate (blue), coumarin 153 (green/yellow) and tris(bipyridine)ruthenium(II) chloride ($Ru(bpy)_3^{2+}$) (red) considering a QY of 0.60, 0.38 and 0.063, respectively.

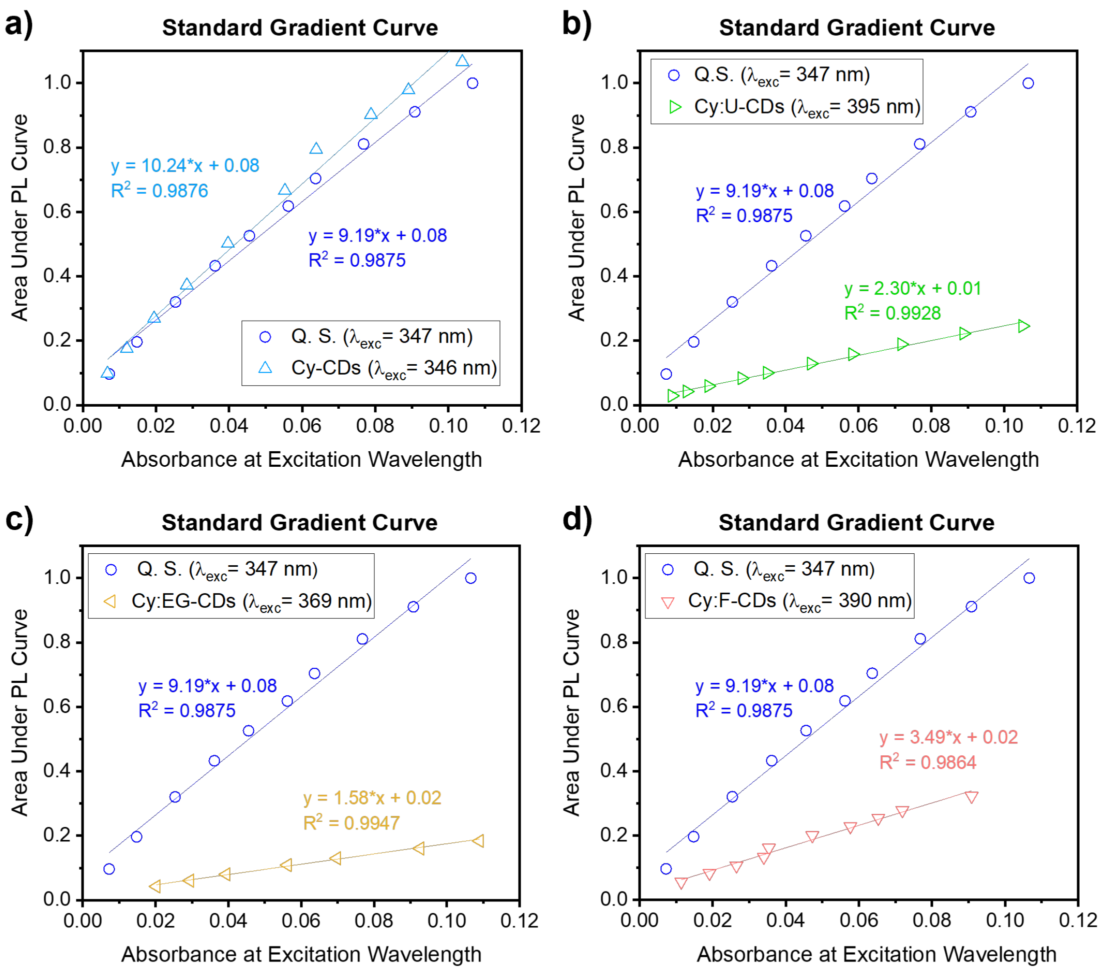

Figure S22. Standard Gradient Curves of CDs for determination of FQY specific to band A. Estimations relative to quinine sulphate (Q.S) standard considering a FQY of 0.60.

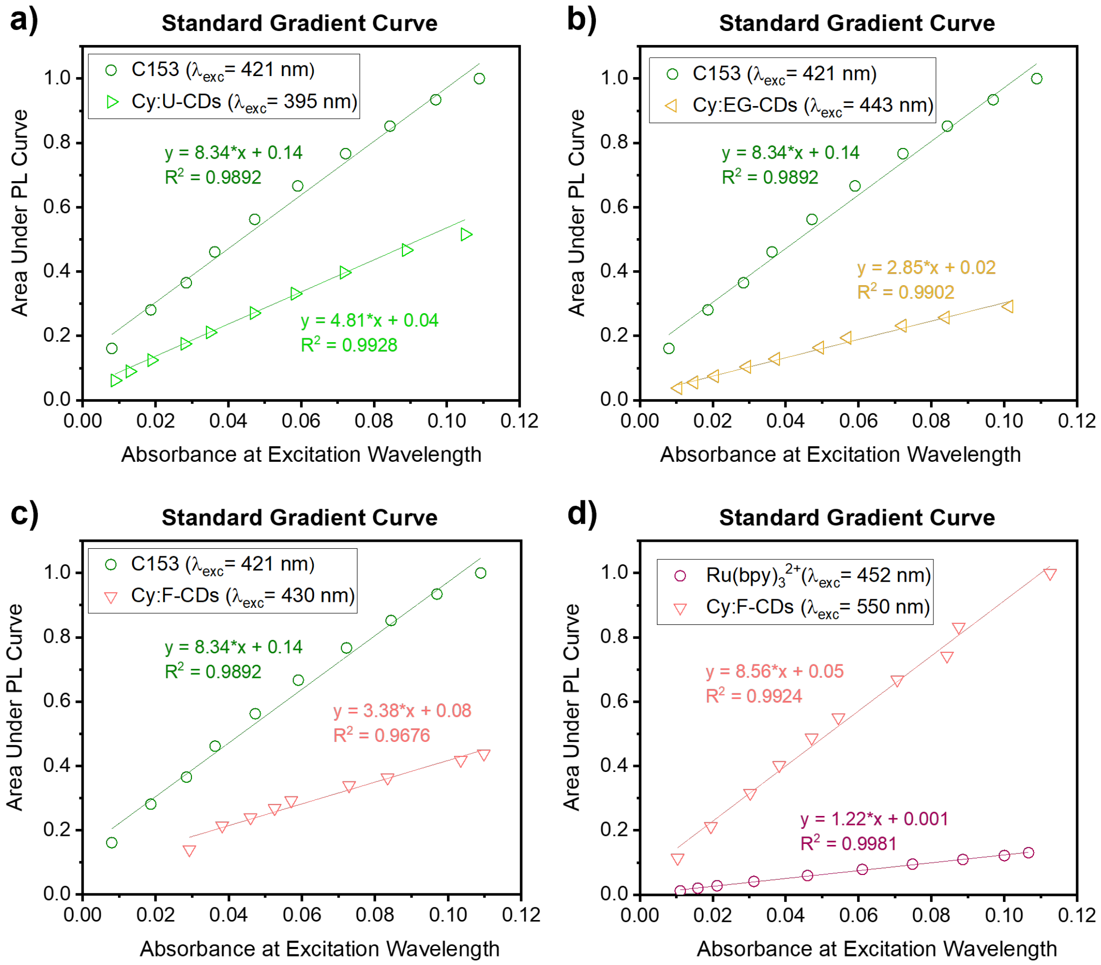

Figure S23. Standard Gradient Curves of CDs for determination of FQY specific to band B (a-c) and C (d). Estimations relative to standards: coumarin 153 (C 153) and tris(bipyridine)ruthenium(II) chloride ($Ru(bpy)_3^{2+}$) considering a FQY of 0.38 and 0.063, respectively.

10. X-ray photoelectron spectroscopy (XPS)

Cy-CDs	Binding Energy Max / eV	Elemental composition / %	Deconvolution Functionality
C 1s	285	27.86	C-C/C=C
C 1s	285.7	12.51	C-COO/S-C
C 1s	286.8	11.81	N-C/O-C
C 1s	289	15.11	O=C/O-C=O
N 1s	400.8	3.82	Amine/Pyrrolic N
N 1s	402.3	0.93	Imide/Graphite N
O 1s	532.2	17.83	C=O/O-C=O/C-OH/C-O-C
O 1s	533.6	6.61	O=C-O
S 2p	163.9	2.16	$2p_{3/2}$ of -C-S- (thiophene)
S 2p	165.1	1.1	$2p_{1/2}$ of -C-S- (thiophene)
S 2p	168.3	0.08	C-SO_2-/C-SO_3-
S 2p	169.5	0.04	Sulphate

Table S2: Narrow XPS peak positions, elemental compositions and functionality. Signals referenced to the hydrocarbon peak at 281.46 eV.

Cy:U-CDs	Binding Energy Max / eV	Elemental composition / %	Deconvolution Functionality
C 1s	285	4.22	C-C/C=C
C 1s	285.9	15.5	C-COO/S-C
C 1s	286.9	13.85	N-C/O-C
C 1s	289.2	22.01	O=C/O-C=O
N 1s	400.7	16.86	Amine/Pyrrolic N
N 1s	402.2	2.7	Imide/Graphite N
O 1s	532.3	19.88	C=O/O-C=O/C-OH/C-O-C
O 1s	534	3.31	O=C-O
S 2p	164.2	0.58	$2p_{3/2}$ of -C-S- (thiophene)
S 2p	165.4	0.3	$2p_{1/2}$ of -C-S- (thiophene)

Table S3: Narrow XPS peak positions, elemental compositions and functionality. Signals referenced to the hydrocarbon peak at 281.42 eV.

Cy:EG-CDs	Binding Energy Max / eV	Elemental composition / %	Deconvolution Functionality
C 1s	285	38.08	C-C/C=C/S-C
C 1s	286.4	19.56	N-C/O-C
C 1s	288	2.02	O=C
C 1s	288.9	8.34	O-C=O
N 1s	400.1	5.49	Amine/Pyrrolic N
N 1s	401.7	1.15	Imide/Graphite N
O 1s	531.1	2.94	C=O/O-C=O
O 1s	532.1	12.31	C-OH/C-O-C
O 1s	533.5	5.7	O=C-O
S 2p	163.7	1.74	$2p_{3/2}$ of -C-S- (thiophene)
S 2p	164.9	0.89	$2p_{1/2}$ of -C-S- (thiophene)
S 2p	168.3	0.25	C-SO_2-/C-SO_3-
S 2p	169.5	0.13	Sulphate

Table S4: Narrow XPS peak positions, elemental compositions and functionality. Signals referenced to the hydrocarbon peak at 281.62 eV.

Cy:F-CDs	Binding Energy Max / eV	Elemental composition / %	Deconvolution Functionality
C 1s	285	41.92	C-C/C=C/S-C
C 1s	286.5	12.26	N-C/O-C
C 1s	287.9	6.52	O=C
C 1s	288.9	4.87	O-C=O
N 1s	398.9	2.18	Amine
N 1s	399.9	7.65	Imide/Minor Graphite N contribution
O 1s	531.1	6.1	C=O/O-C=O
O 1s	532	9.59	C-OH/C-O-C
O 1s	533.4	3.91	O=C-O
S 2p	163.5	0.22	$2p_{3/2}$ of -C-S- (thiophene)
S 2p	164.7	0.11	$2p_{1/2}$ of -C-S- (thiophene)
S 2p	168.2	0.35	C-SO_2-/C-SO_3-
S 2p	169.4	0.18	Sulphate

Table S5: Narrow XPS peak positions, elemental compositions and functionality. Signals referenced to the hydrocarbon peak at 281.58 eV.

Interestingly, a comparative analysis of the XPS spectra for all the different samples highlights the variations in functional group ratio between **Cy-CDs** and co-doped CDs. In the case of **Cy:U-CDs**, which have a 4 fold higher N content than **Cy-CDs**, showed a 0.16 ratio of imine/graphite N to amine/pyrrolic N, which is 1.5 times smaller than the ratio seen for **Cy-CDs** (Table S3). The ratio of O=C-O to C=O/O–C=O/C–OH/C–O–C also decreased by a factor of 2.2 for **Cy:U-CDs** (Table S3), whereas the ratio between S containing groups was kept the same. These differences in heteroatom functionality correlate to the differences observed for the C 1s deconvoluted signals for **Cy:U-CDs** (Table S3), which in turn indicate an increase in

the N–C/O–C and O=C/O–C=O content by 1.6 and 2.3 fold, respectively, as expected from the excess of urea **3** used within the synthesis. Comparing **Cy:EG-CDs** to **Cy-CDs** (Tables S2 vs S4) showed a 1.9-fold increment of the N–C/O–C band to the rest of the functional groups in the C 1s signal, unlike the O=C/O–C=O band which was reduced around 2.1 times. The most notable aspect of the O 1s analysis is the fitting of the O 1s signal, revealing a larger contribution of C–OH/C–O–C than that of C=O/O–C=O groups. The fitted functionalities in the N 1s signal showed that N composition variations between both CDs were negligible. It is worth noting, that a 3.7-fold increment of the C–SO_2–/C–SO_3– and sulphate groups was observed as per the S 2p deconvolution data (**Cy:EG-CDs**, Table S4). Finally, it is apparent from the high-resolution XPS data for **Cy:F-CDs**, that using formamide **5** as the solvent has a significant effect on modifying the CD composition. In this case, the dominant N 1s peak (Table S5) can be assigned to an imide/graphitic N which is 3.5 times larger than the amine contribution. These results agree with the observed increment of the O 1s band at 531.1 eV which correlates to C=O groups in imide functionalities. Moreover, the –C–S– ($2p_{3/2}$, thiophene) and –C–S– ($2p_{1/2}$, thiophene) contributions in the S 2p scan are negligible in the overall composition for this material. Taken together, these results suggest that formamide **5** is able to replace **2** as the major doping agent during the synthesis of **Cy:F-CDs**.

11. Biomarkers for detection of brain cancer

NMR characterization of DBCO-CD (DBCO-**Cy:U-CDs*****)***

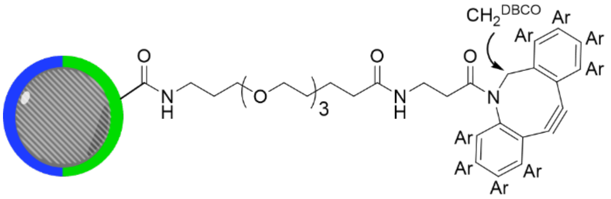

^{1}H NMR (500 MHz, D_2O) characteristic signals, δ 7.50 – 6.72 (m, Ar), 4.85 (m, CH_2^{DBCO}a) 3.70 – 2.50 (PEG linker and CH_2^{DBCO}b); **^{13}C NMR** (126 MHz, D_2O) δ 173.8, 161.5, 159.6, 150.7, 147.7, 132.0 (Ar), 129.1 (Ar), 128.1 (Ar), 126.9 (Ar), 125.6 (Ar), 122.4, 121.5, 114.7, 107.9, 69.6, 69.5, 69.5, 69.3, 69.3, 68.5, 68.5, 68.3, 62.5, 55.2 (CH_2^{DBCO}), 42.4, 42.1, 41.3, 40.3, 40.0, 39.1, 38.8, 37.5, 36.9, 36.3, 34.8, 29.1, 29.1, 28.3, 10.5.

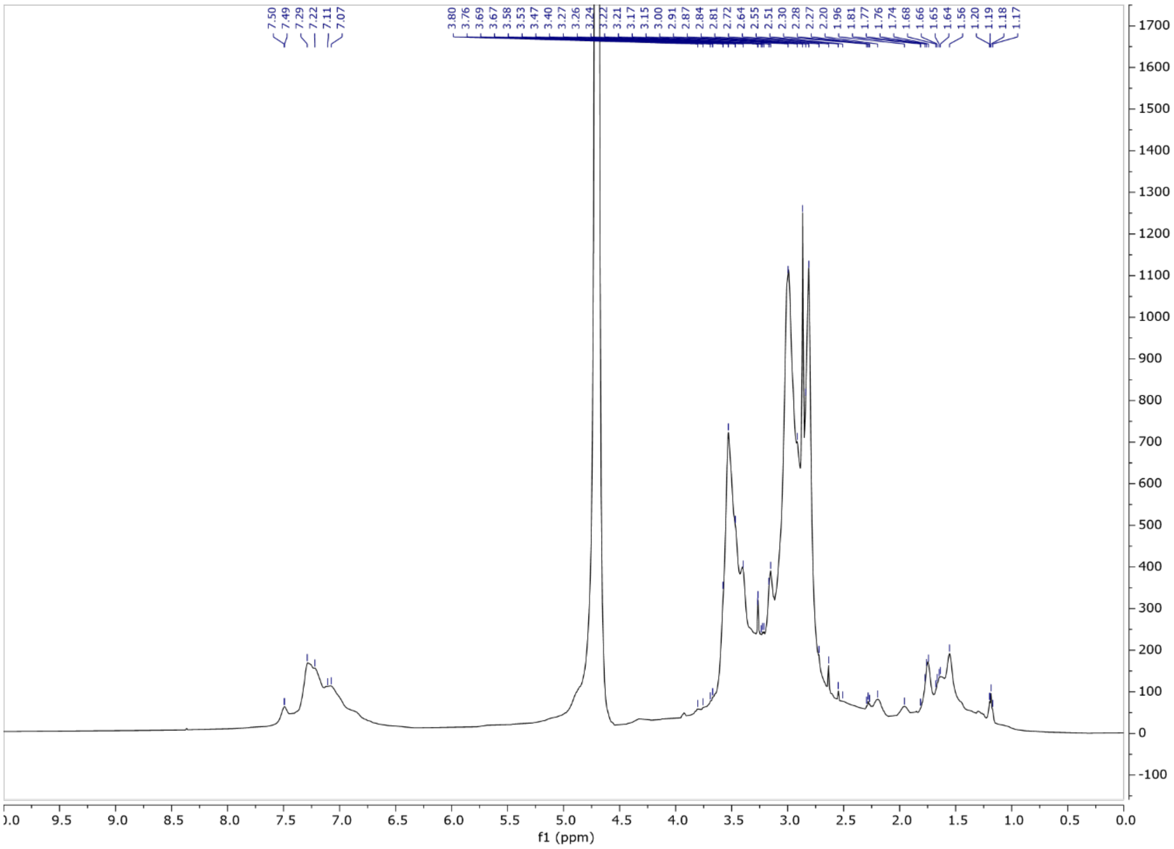

Figure S24. ^{1}H NMR spectrum of **DBCO-CDs** (D_2O, 500 MHz).

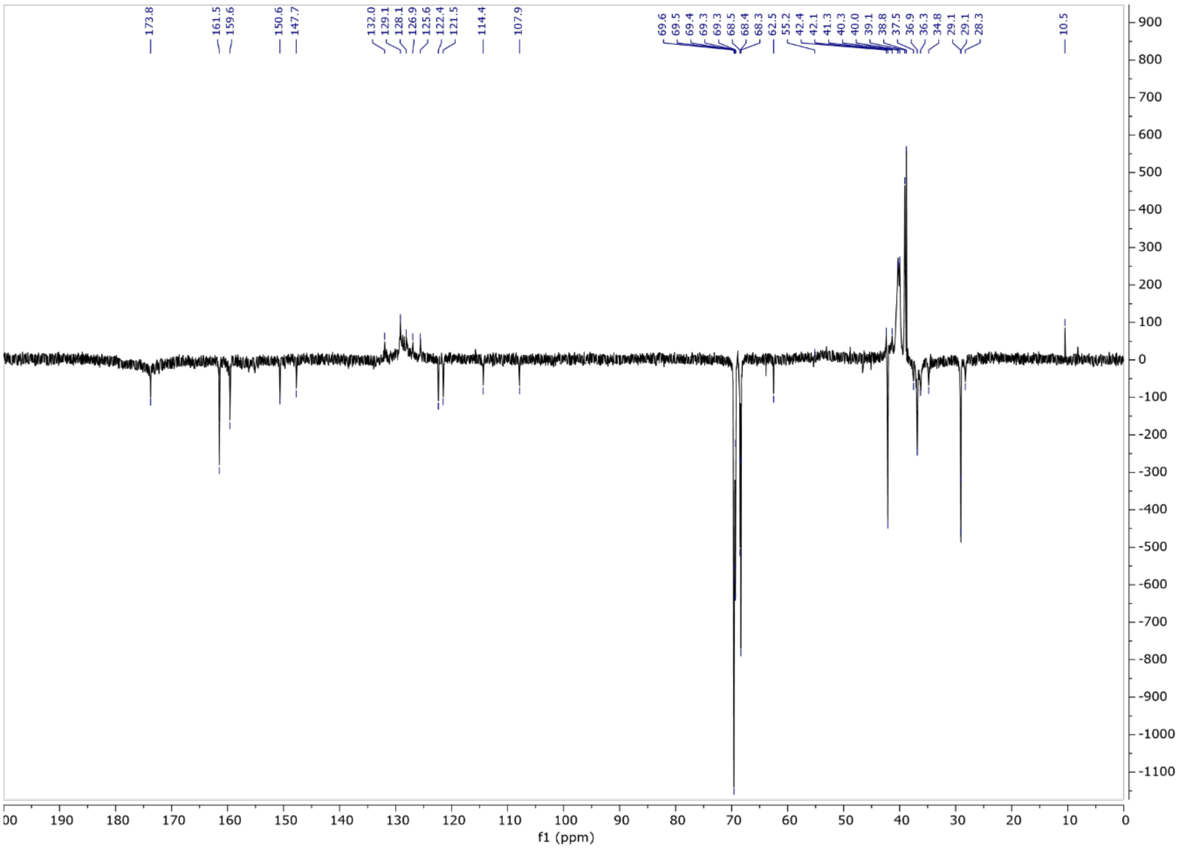

Figure S25. ^{13}C APT NMR spectrum of **DBCO-CDs** (D_2O, 126 MHz).

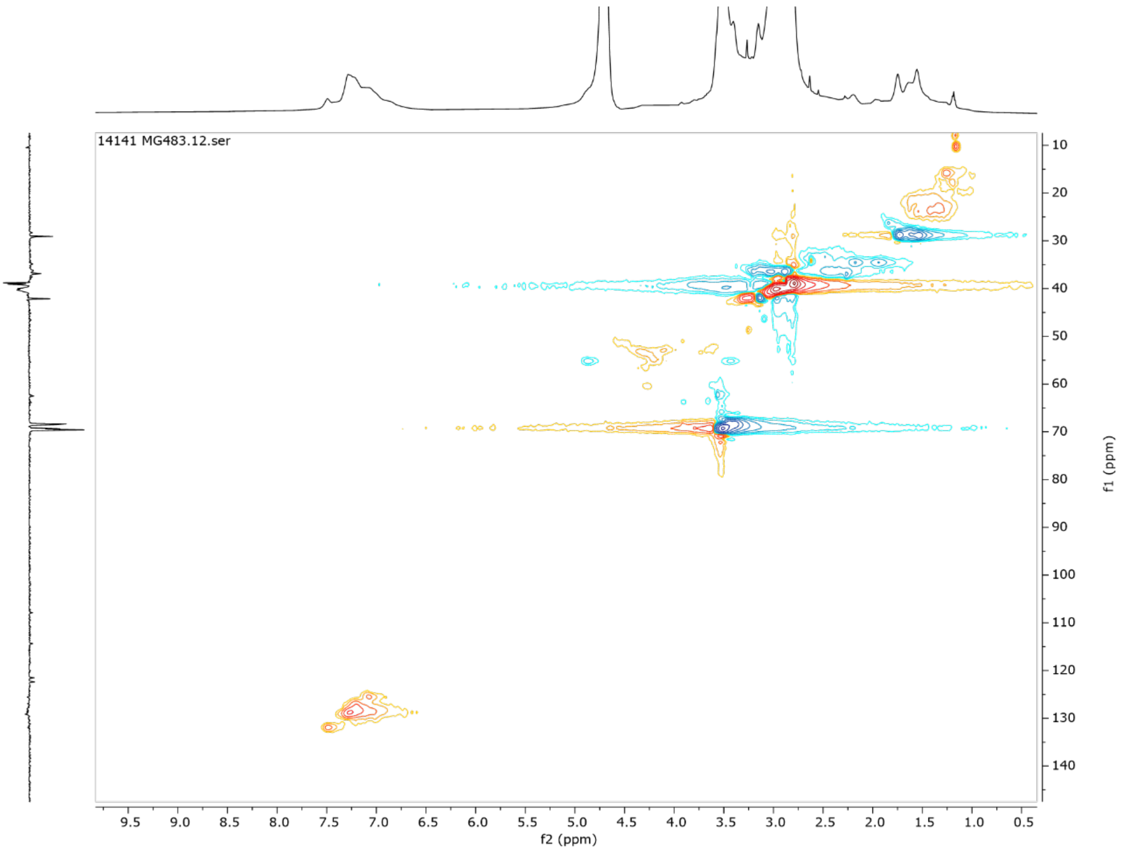

Figure S26. ${}^1H$-${}^{13}C$ HSQC NMR spectrum of **DBCO-CDs** (D_2O, 500 MHz).

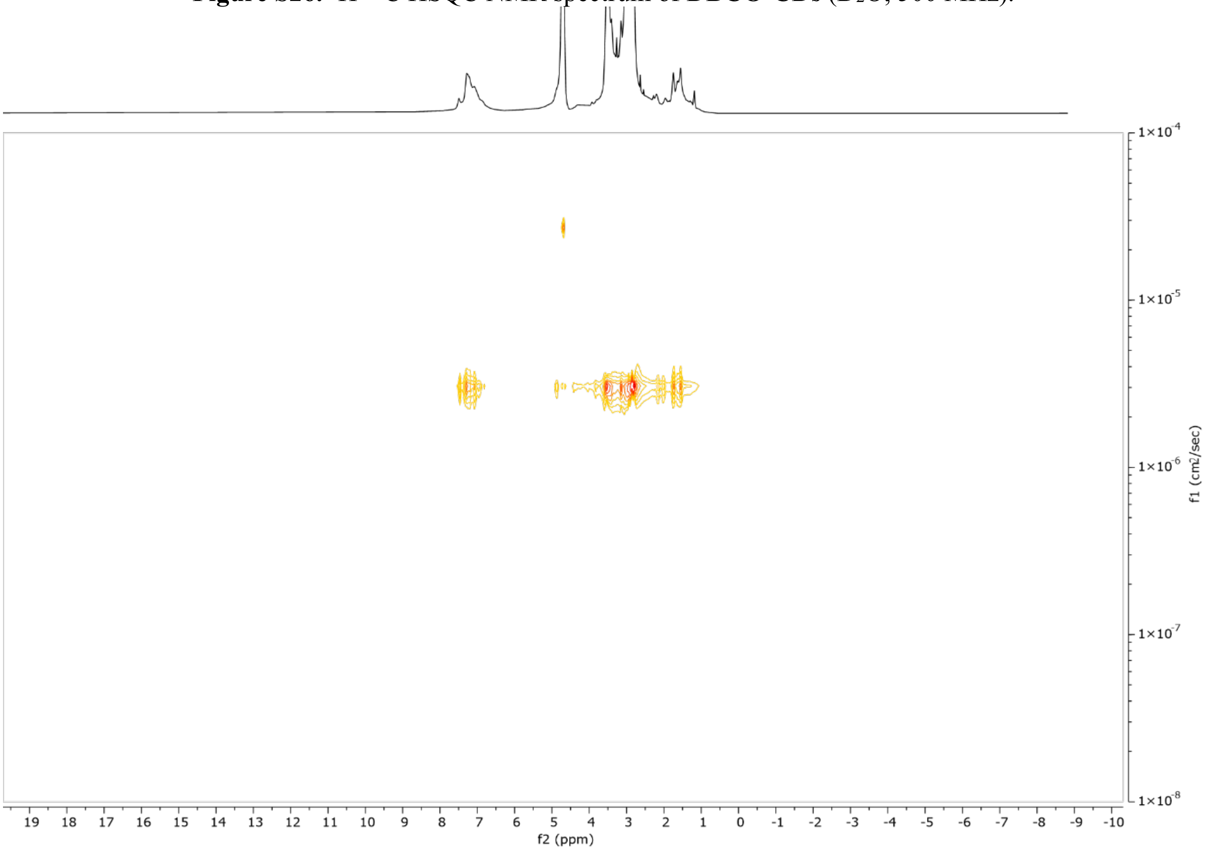

Figure S27. ${}^1H$ DOSY NMR of **DBCO-CDs** (D_2O, 500 MHz).

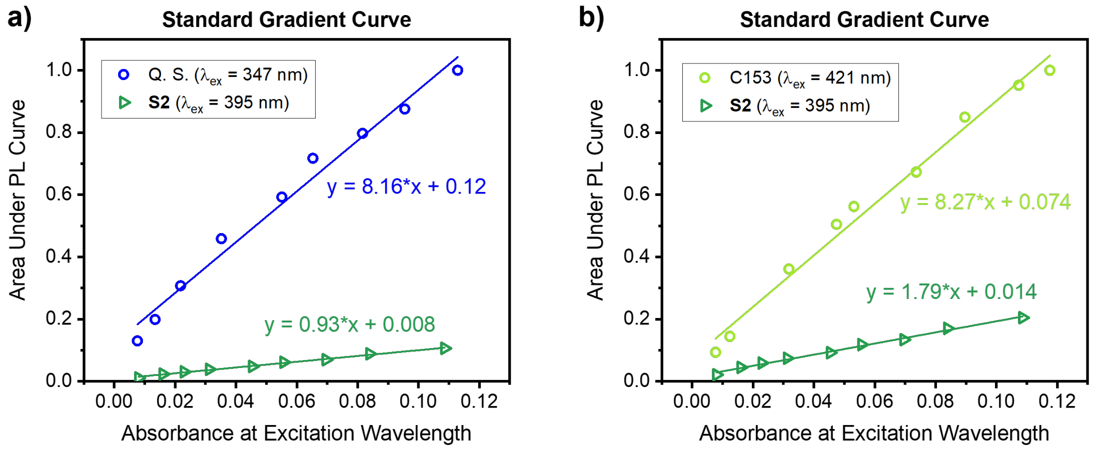

Figure S28. Standard Gradient Curves of CD-DBCO (**S2**) for determination of FQY specific to band A (450 nm) and band B (499 nm). FQY were found to be 0.07 and 0.08., respectively. Estimations relative to quinine sulphate (Q.S) standard considering a FQY of 0.60.

Ab-CDs conjugation

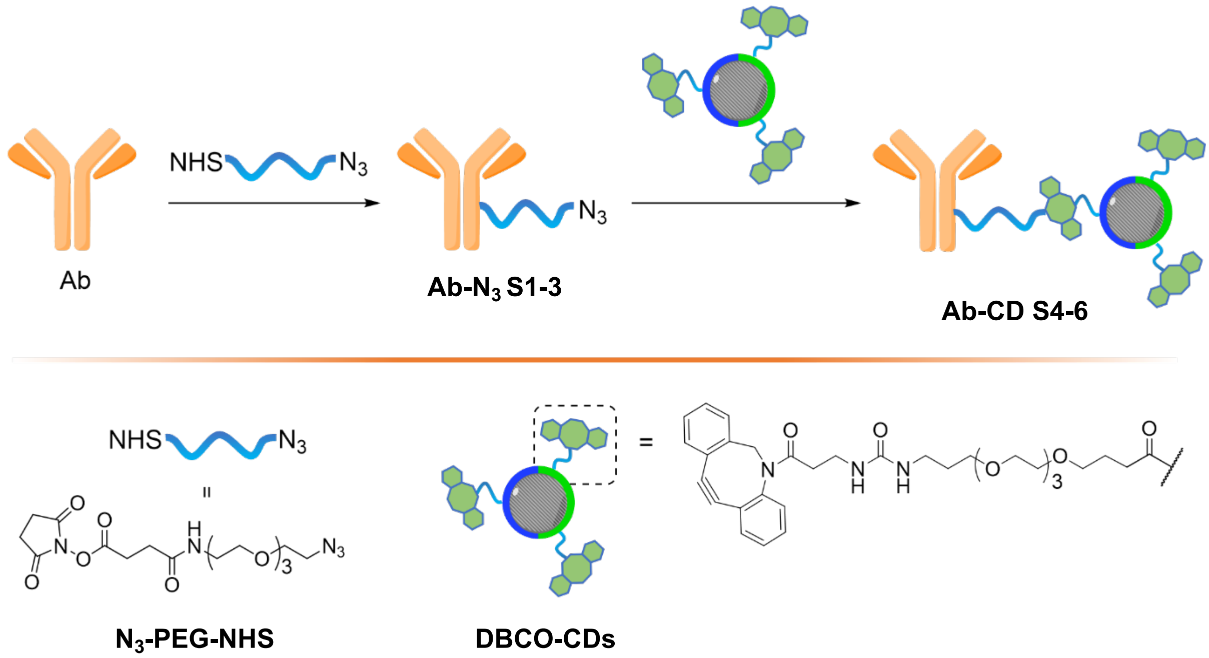

Scheme S1. General synthetic pathway to obtain **Ab-CD S4-6**

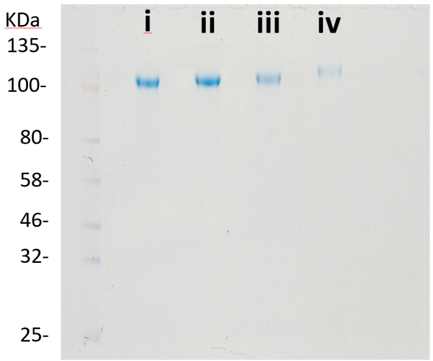

Figure S29. NuPAGE Gel electrophoresis of Ab-CD derivatives **S4-6**: i) native Abs, ii) **S4** iii) **S5**, iv) **S6**.

Tissue staining

Figure	Patient ID	Age	Sex	Site	Diagnosis	WHO grade	Legacy #
**5(b,c), S26**	08/0057B	58	M	n.s.	GBM	4	08N90026183
**S27**	08/0160B	55	M	right hemisphere	GBM	4	08N90026275
**5(a)**	21/N1214A1	76	M	spinal L5/s1	schwannoma	1	not stated

Table S6: Clinical data for brain cancer tumour cells stained with **Ab-CDs** biomarker. IDH status not stated.

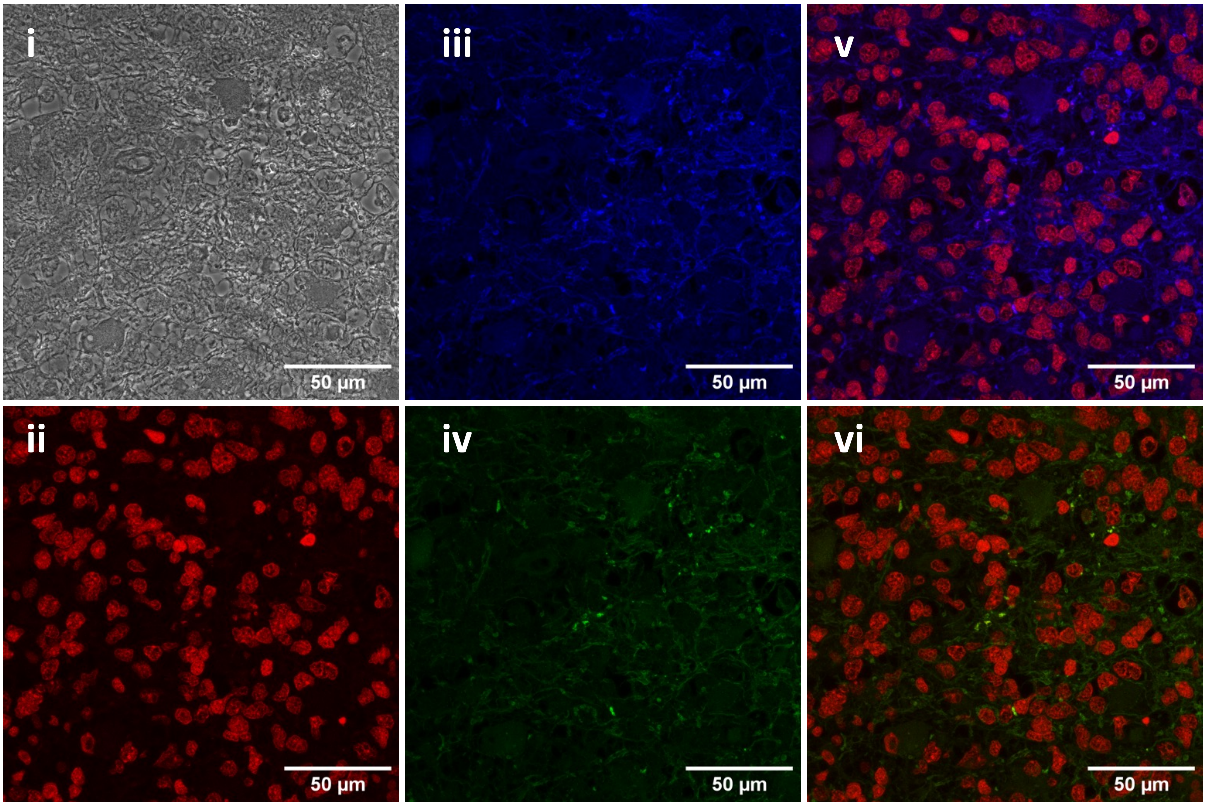

Figure S30. Confocal microscope images of malignant brain tumour glioblastoma stained with **Ab-CDs** (patient ID: 08/0057B). Under bright field (i) and the blue laser excitation: immunofluorescence of red labelled nuclei (ii), fluorescent channel at 450 nm (iii), fluorescent channel at 500 nm (iv) and merge microscopes of the immunostained nuclei with the blue or the green channels (v and vi, respectively).

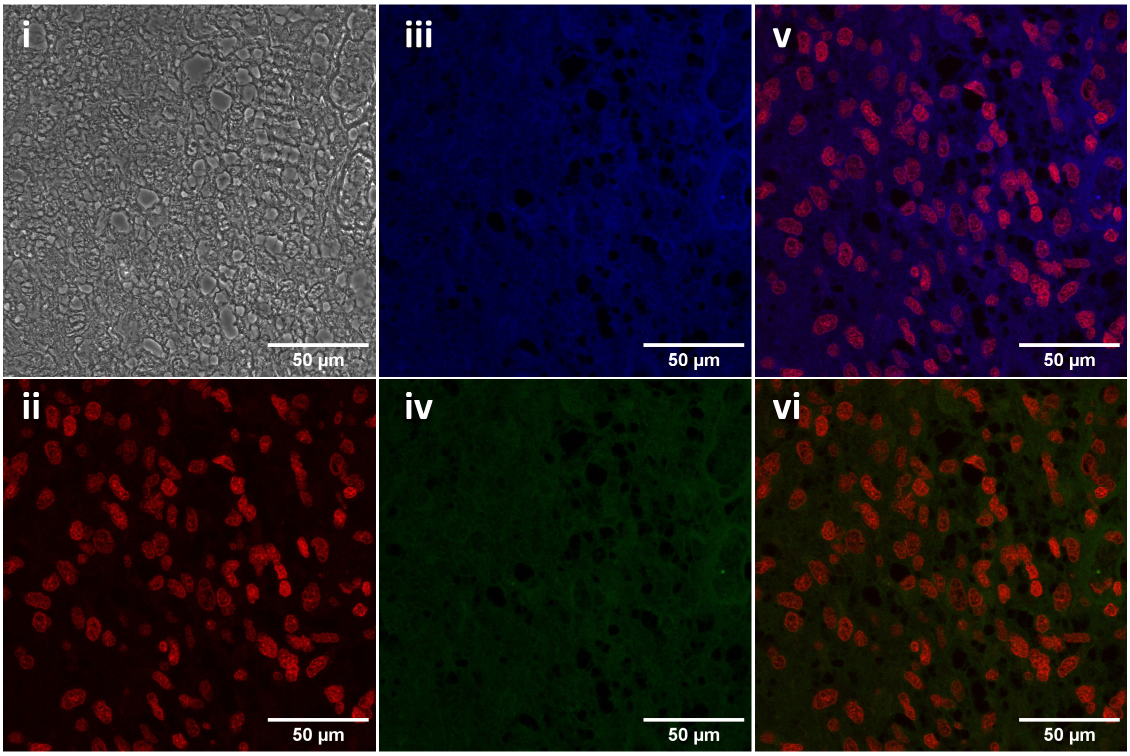

Figure S31. Confocal microscope images of malignant brain tumour glioblastoma stained with **Ab-CDs** (patient ID: 08/0160B). Under bright field (i) and the blue laser excitation: immunofluorescence of red labelled nuclei (ii), fluorescent channel at 450 nm (iii), fluorescent channel at 500 nm (iv) and merge microscopes of the immunostained nuclei with the blue or the green channels (v and vi, respectively).

Confocal imaging was carried out by Dr Katy Jepson in the Wolfson Bioimaging Facility on a Leica SP8 AOBS confocal laser scanning microscope attached to a Leica DMi8 inverted epifluorescence microscope using a HC PL APO CS2 63x/1.40 OIL lens at 2x zoom. Images were processed using Fiji (ImageJ).

Image analysis of blue:green CD-labelling intensity

Fluorescence Intensity (FI)	FI (Blue)	FI (Green)	FI Blue:Green ratio
Untreated tissue sample			
Patient ID: 08/0057B n=3	25.2±2.6	7.1±0.6	3.6
Ab-CD labelled samples			
Patient ID: 21/N1214A1), negative control n=3	24.5±3.7	9.4±1.8	2.7
Patient ID: 08/0057B), positive control n=6	43.7±7.1	15.9±2.9	2.7
Patient ID: 08/0160B), positive control n=3	40.9±8.9	15.0±2.8	2.66

The fluorescence intensity values were calculated using FIJI IMAGEJ as follows:

- For each image: Image-> Color -> Split channels
- On each channel: Stk -> Z project (max intensity) all levels
- On the stacked image: Analyze -> Measure
- The value "Mean" is the one reported in the table.

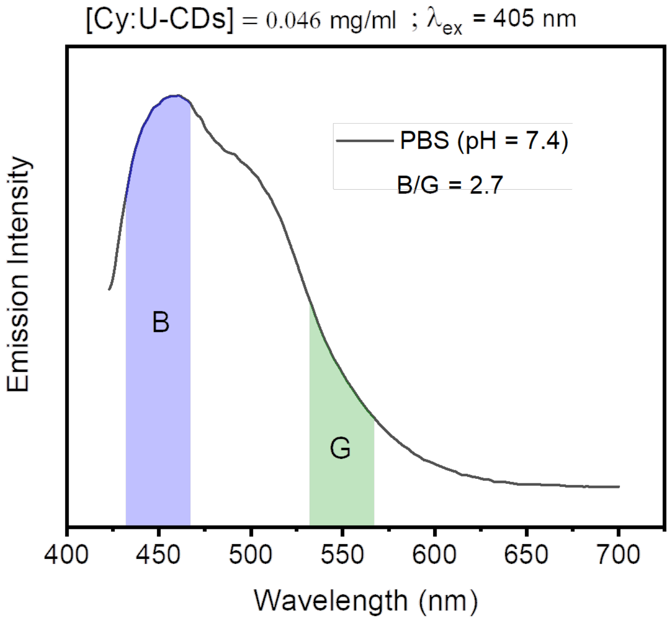

The blue:green fluorescence emission ratio upon excitation at 405 nm as measure above for **Cy-U-CDs** correlates with the values observed for **Ab-CD** labelled samples.

References

[1] Albert M Brouwer. Standards for photoluminescence quantum yield measurements in solution (IUPAC technical report). Pure and Applied Chemistry, 83(12):2213–2228, 2011.

[2] Wang, H. X.; Yang, Z.; Liu, Z. G.; Wan, J. Y.; Xiao, J.; Zhang, H. L., Facile Preparation of Bright-Fluorescent Soft Materials from Small Organic Molecules. *Chemistry* **2016,** *22* (24), 8096-104.

[3] Ghirardello, M.; Shyam, R.; Liu, X.; Garcia-Millan, T.; Sittel, I.; Ramos-Soriano, F. J.; Kurian, K.; Galan, M. C., Carbon Dot-based Fluorescent Antibody Nanoprobes as Brain Tumour Glioblastoma Diagnostics. Nanoscale Adv. **2022**, 4, 1770.
